# Discovery of a novel *Wolbachia* in *Heterodera* expands nematode host distribution

**DOI:** 10.3389/fmicb.2024.1446506

**Published:** 2024-09-25

**Authors:** Taranjot Kaur, Amanda M.V. Brown

**Affiliations:** Department of Biological Sciences, Texas Tech University, Lubbock, TX, United States

**Keywords:** bioinformatics data mining, tylenchid, *Heterodera*, plant-parasitic nematode, endosymbiont, *Wolbachia*, comparative genomics, phylogenomics

## Abstract

Bioinformatics sequence data mining can reveal hidden microbial symbionts that might normally be filtered and removed as contaminants. Data mining can be helpful to detect *Wolbachia*, a widespread bacterial endosymbiont in insects and filarial nematodes whose distribution in plant-parasitic nematodes (PPNs) remains underexplored. To date, *Wolbachia* has only been reported a few PPNs, yet nematode-infecting *Wolbachia* may have been widespread in the evolutionary history of the phylum based on evidence of horizontal gene transfers, suggesting there may be undiscovered *Wolbachia* infections in PPNs. The goal of this study was to more broadly sample PPN *Wolbachia* strains in tylenchid nematodes to enable further comparative genomic analyses that may reveal *Wolbachia’s* role and identify targets for biocontrol. Published whole-genome shotgun assemblies and their raw sequence data from 33 *Meloidogyne* spp. assemblies, seven *Globodera* spp. assemblies, and seven *Heterodera* spp. assemblies were analyzed to look for *Wolbachia*. No *Wolbachia* was found in *Meloidogyne* spp. and *Globodera* spp., but among seven genome assemblies for *Heterodera* spp., an *H. schachtii* assembly from the Netherlands was found to have a large *Wolbachia-*like sequence that, when re-assembled from reads, formed a complete, circular genome. Detailed analyses comparing read coverage, GC content, pseudogenes, and phylogenomic patterns clearly demonstrated that the *H. schachtii Wolbachia* represented a novel strain (hereafter, denoted *w*Het). Phylogenomic tree construction with PhyloBayes showed *w*Het was most closely related to another PPN *Wolbachia, w*Tex, while 16S rRNA gene analysis showed it clustered with other *Heterodera Wolbachia* assembled from sequence databases. Pseudogenes in *w*Het suggested relatedness to the PPN clade, as did the lack of significantly enriched GO terms compared to PPN *Wolbachia* strains. It remains unclear whether the lack of *Wolbachia* in other published *H. schachtii* isolates represents the true absence of the endosymbiont from some hosts.

## Introduction

Microbial symbioses are essential components of ecosystems that play major roles in evolution and ecology. These symbiotic interactions include mutualism, parasitism, and commensalism (Fransolet et al., 2012; Goffredi, 2010; Lamelas et al., 2011; Lefoulon et al., 2016; Moya et al., 2014). The bacterial endosymbiont *Wolbachia* is widespread in terrestrial arthropods, infecting approximately 40% of terrestrial arthropod species (Zug and Hammerstein, 2012), and is almost universally present in filarial nematodes as an obligate mutualist (Lefoulon et al., 2016; Slatko et al., 2010; Taylor et al., 2013). It has been discovered in several plant-parasitic nematodes (hereafter, PPNs) (Brown, 2018; Brown et al., 2016; Haegeman et al., 2009; Kaur and Brown, 2024). *Wolbachia* strains vary in role and can range from parasitic to mutualistic with their hosts (Gill et al., 2014). Except for filarial nematodes, few other nematodes have been reported to harbor *Wolbachia*, with only a few tylenchid reported as *Wolbachia* hosts: *Radopholus similis* (Haegeman et al., 2009)*, Radopholus arabocoffeae* (Haegeman et al., 2009)*, Pratylenchus penetrans* (Brown, 2018; Denver et al., 2016), a fourth PPN nematode (Weyandt et al., 2022), and an entomopathogenic tylenchid, *Howardula* sp. (Dudzic et al., 2022). However, it is unclear whether the apparent rarity of *Wolbachia* in non-filarial nematodes, including PPNs, is just a result of undersampling. Interestingly, a recent highly sensitive PCR-based survey suggests that *Wolbachia* may be widespread in other nematodes (Kaur and Brown, 2024).

It has been hypothesized that nematode-infecting *Wolbachia* may have been widespread at one point as there is evidence of horizontal gene transfers leaving *Wolbachia*-like fragments in nematode genomes across the nematode phylogeny (Dunning Hotopp, 2011; Husnik and McCutcheon, 2017; Koutsovoulos et al., 2014; McNulty et al., 2010). Furthermore, past phylogenomic analyses placed PPN *Wolbachia* strains at the origin of the *Wolbachia* phylogeny, at the base of other supergroups (Brown et al., 2016; Scholz et al., 2020) and with long branches suggesting longstanding symbioses in the order Tylenchida (Brown et al., 2016; Dudzic et al., 2022) and supporting *Wolbachia*’s emergence early in the diversification of terrestrial Ecdysozoa hosts, during a time of expanding plant-diet specialization. The conservation of iron/heme biosynthesis and other functions at the root of the clade also suggests a potential facultative nutritional mutualism (Brown et al., 2016; Weyandt et al., 2022). Whereas extant *Wolbachia* strains from arthropods can cause host reproductive manipulations such as cytoplasmic incompatibility (CI), parthenogenesis induction, male-killing, or genetic male feminization (Bing et al., 2023; Correa and Ballard, 2016; Sanaei et al., 2020; Werren et al., 2008; Werren, 1997; Zhu et al., 2020), *Wolbachia* strains from filarial nematodes and several other hosts act as beneficial or obligate mutualists (Hosokawa et al., 2010; Lefoulon et al., 2016). To date, however, the role of PPN *Wolbachia* remains unclear.

Among the PPNs, which are estimated to cost approximately ~$100 billion in global crop losses annually (Nicol et al., 2011), some of the most devastating species (Jones et al., 2013) have been found to host *Wolbachia*, raising interest in potential future *Wolbachia*-based controls. To evaluate this potential, it is important to understand the prevalence, diversity, and distribution of PPN *Wolbachia*. An early study of *Wolbachia* in *Radopholus similis* showed 100% prevalence, which might indicate a mutualist role for this strain (Haegeman et al., 2009). However, a more recent genome data mining study showed a discontinuous distribution of *Wolbachia* in *Radopholus* sp. and *Pratylenchus* spp. across North America, South America, and Africa, suggesting a non-obligatory role in these hosts (Wasala et al., 2023). The presence of the *Wolbachia* strain *w*Ppe in *Pratylenchus* spp. was positively correlated with female-biased sex ratios, suggesting a potential role in host sex ratio modulation via an unknown mechanism (Wasala et al., 2019). Yet, with few PPN *Wolbachia* characterized to date, it is difficult to interpret these patterns.

Therefore, to understand the prevalence, diversity, and role of *Wolbachia* in PPNs, we searched genomic sequence data from public databases for hidden *Wolbachia* sequences, focusing on the top three most damaging PPNs, namely, *Meloidogyne* spp., *Globodera* spp., and *Heterodera* spp. (Jones et al., 2013). We screened whole-genome shotgun (WGS) assemblies and their sequence read archive (SRA) and recovered a new *Wolbachia* genome from a *Heterodera schachtii* WGS assembly (designated as *w*Het). We analyzed its phylogenetic place and annotated its genomic features and found functional enrichment suggesting it may be a facultative mutualist with a role in heme homeostasis.

## Materials and methods

### SRA data screening

We downloaded WGS assemblies and their SRA reads for *Meloidogyne* spp. (33 WGS assemblies), *Globodera* spp. (seven WGS assemblies), and *Heterodera* spp. (seven WGS assemblies). The SRA data and the assemblies were analyzed for the presence of *Wolbachia*-like sequences using a two-step approach with blastn in Blast+ v2.10.1 (Camacho et al., 2009). In the first step, SRA data and the assemblies were compared to a custom database of *Wolbachia* genome sequences using blastn. This custom database was made by compiling known PPN *Wolbachia* genomic sequences into a single file and using makeblastdb to make these a nucleotide sequence reference database to search against using blastn. In the second step, the blast hits from first step were used as queries for the second blastn analysis against the complete nt reference database from NCBI. Contigs were classified as plant-parasitic nematode (PPN) *Wolbachia* if their top blast hits showed a closer match to *Wolbachia* strains from PPN (*w*Ppe, *w*Tex, Pp_GH2, Pp_Cr, Rs_N1, Rs_14, Rs_5) compared to other non-plant-parasitic nematode strains.

### Detection of nematode hosts

To confirm the host nematode species and look for contaminating nematodes or cryptic species in the assembly, blastn against a custom nematode COI gene database was performed.

### Genome annotation and polishing by read mapping

The coverage of the *Wolbachia*-matching regions and flanking regions was assessed using the pileup.sh script^1^ from SRA Nanopore DNA reads derived from the same project as the *Wolbachia*-containing WGS assembly (NCBI accession PRJNA767548; reads SRR16146220-SRR16146534 and SRR16675965-SRR16675966 and SRR28675229-SRR28675543) mapped to the WGS *Wolbachia* scaffold using the BWA software package with the BWA-MEM algorithm (Li and Durbin, 2009). Raw reads that mapped to *Wolbachia* (as a sam file) were visualized in Geneious Prime and then, removing the WGS-derived *Wolbachia* reference, the reads spanning gap regions were assembled *de novo* and the final consensus contig was used to extend and fill-in the ends of the sequence containing uncalled bases (N’s) in the junctions between *Wolbachia* and non-*Wolbachia* regions of the contigs to assess a possible overlap that would create a circular genome with overlapping ends. CheckM, which looks for the genes in the genome using hmmer (Finn et al., 2011; Parks et al., 2015), was used to analyze the completeness and contamination of the assembled genome. In addition, BUSCO was also used to compute the completeness of the assembled genome (Manni et al., 2021). Prokka 1.14.6 was used to annotate the genes in the endosymbiont assembly as well as the genomes used for comparative genomics (Seemann, 2014).

### Comparative genomics and pangenome analysis

Genome assemblies of *Wolbachia* strains from insects, filarial nematodes, and PPNs were downloaded from NCBI, for comparison to the newly discovered *Wolbachia* strain. Representatives included published *Wolbachia* genomes from supergroups A, B, C, D, E, F, J, M, S, T, and V and supergroup L (Table 1) and an out-group *Candidatus* Mesenet longicola.

**Table 1:** Description of genomes used for comparative genomics and NCBI accession numbers.

NCBI accession no.	*Wolbachia* strain (Host name)	%GC	#contigs	Length (Mb)	Complete/draft genomes
SUPERGROUP A					
CP101657.1	*w*AlbA (*Aedes albopictus*)	0.354	1	1.19093	Complete
NZ_ACFP01000256.1	*w*Uni (*Muscidifurax uniraptor*)	0.3516	284	0.866349	Draft
NC_021089.1	*w*Ha (*Drosophila simulans*)	0.3509	1	1.295804	Complete
CP041215.1	*w*CauA (*Carposina sasakii*)	0.3499	1	1.449344	Complete
CP046925.1	*w*Mel (*Drosophila melanogaster*)	0.3523	1	1.267783	Complete
LK055284.1	*w*Au (*Drosophila simulans*)	0.3522	1	1.268461	Complete
NZ_JAATLB010000020.1	*w*Bic (*Drosophila bicornuta*)	0.351	1	1.182871	Complete
CP001391.1	*w*Ri (*Drosophila simulans*)	0.3516	1	1.445873	Complete
NZ_CP042904.1	*w*Ana (*Drosophila ananassae*)	0.3519	1	1.40146	Complete
SUPERGROUP B					
CP031221.1	*w*AlbB (*Aedes albopictus*)	0.3443	1	1.484007	Complete
CP016430.1	*w*Bta (*Bemisia tabaci*)	0.339	31	1.306495	Draft
CP003883.1	*w*No (*Drosophila simulans*)	0.3401	1	1.301823	Complete
NC_010981.1	*w*Pip (*Culex quinquefasciatus*)	0.3419	2	1.482455	Complete
NZ_AERW01000001.1	*w*VitB (*Nasonia vitripennis*)	0.3399	509	1.105401	Draft
CP021120.1	*w*Meg (*Chrysomya megacephala*)	0.3395	1	1.376868	Complete
NZ_CP084694.1	*w*AnD (*Anopheles demeilloni*)	0.3358	1	1.231247	Complete
SUPERGROUP C					
NC_018267.1	*w*Oo (*Onchocerca ochengi*)	0.3207	1	0.95799	Complete
HG810405.1	*w*Ovc (*Onchocerca volvulus*)	0.3207	1	0.960618	Complete
NZ_CP046578.1	*w*Dim (*Dirofilaria immitis*)	0.327	1	0.920122	Complete
SUPERGROUP D					
AE017321.1	*w*Bm (*Brugia malayi*)	0.3418	1	1.080084	Complete
CP046577.1	*w*Lsig (*Litomosoides sigmodontis*)	0.3212	1	1.045802	Complete
NJBR02000001.1	*w*Wb (*Wuchereria bancrofti*)	0.3434	104	1.06085	Draft
SUPERGROUP E					
CP015510.2	*w*Fol (*Folsomia candida*)	0.3435	1	1.801626	Complete
SUPERGROUP F					
AP013028.1	*w*Cle (*Cimex lectularius*)	0.3625	1	1.25006	Complete
CP116768.1	*w*CfeJ (*Ctenocephalides felis*)	0.3557	1	1.20178	Complete
SUPERGROUP J					
NZ_CP046579.1	*w*Ctub (*Cruorifilaria tuberocauda*)	0.3228	1	0.863988	Complete
NZ_CP046580.1	*w*Dcau (*Dipetalonema caudispina*)	0.2822	1	0.863427	Complete
SUPERGROUP L					
NZ_MJMG01000001.1	*w*Ppe (*Pratylenchus penetrans*)	0.3216	36	0.975127	Draft
JAIXMJ010000001.1	*w*Tex (unknown PPN)	0.3349	192	1.012782	Draft
SRR26324238	Pp_Cr (*Pratylenchus penetrans*)	0.3238	606	0.971259	Draft
SRR26324233	Pp_GH2 (*Pratylenchus penetrans*)	0.3256	90	1.030112	Draft
SRR26324215	Rs_14 (*Radopholus similis*)	0.3282	99	0.956972	Draft
SRR26324217	Rs_5 (*Radopholus similis*)	0.3299	62	0.927058	Draft
SRR26324214	Rs_N1 (*Radopholus similis*)	0.3331	68	0.95737	Draft
SUPERGROUP M					
NZ_JACVWV010000040.1	*w*Pni (*Pentalonia nigronervosa*)	0.3409	187	1.457187	Draft
SUPERGROUP S					
NZ_WQMQ01000001.1	*w*Apolv1K5 (*Atemnus politus*)	0.3561	373	1.445964	Draft
JAAXCS010000001.1	*w*Apolv1K3 (*Atemnus politus*)	0.3551	200	1.404177	Draft
SUPERGROUP T					
NZ_CP061738.1	*w*Chem (*Cimex hemipterus*)	0.3537	34	1.291339	Draft
SUPERGROUP V					
CP051156.1	*w*CfeT (*Ctenocephalides felis*)	0.3518	1	1.495538	Complete
SUPERGROUP W					
CP092368.1	*w*How (*Howardula* sp.)	0.2953	1	0.553558	Complete

To detect orthologs, we used Roary (Page et al., 2015), and pangenome and core genome comparisons were performed based on the gene_presence_absence.csv file obtained in Roary outputs, depicted using the online Venn drawing tool.^2^ Venn diagrams were made to identify the overlap and differences between the gene sets and infer the biological significance of shared and unique genes in the new *Wolbachia* strain compared to other PPN *Wolbachia* genomes and *Wolbachia* genomes from filarial nematodes and insects.

Genomic features, such as genome size, number of proteins versus hypothetical proteins, number of tRNA and rRNA genes, % GC content, number of pseudogenes, number of ankyrin genes, transposases, phage-related genes, and coding density percent, of new *Wolbachia* strain were compared to members of supergroup L, including *w*Ppe (from *Pratylenchus penetrans*, Oregon, USA), Pp_Cr (from *Pratylenchus penetrans*, Costa Rica), Pp_GH2 (from *Pratylenchus penetrans*, Oregon, USA), Rs_N1 (from *Radopholus similis*, Nigeria), Rs_14 (from *Radopholus similis*, Colombia), Rs_5 (from *Radopholus similis*, Uganda), *w*Tex (from unknown PPN, predicted *Helicotylenchus* sp.), and *w*Mel (from *Drosophila melanogaster*). Pseudofinder was used to detect the pseudogenes and evolutionary interference in endosymbiont genomes (Syberg-Olsen et al., 2022). The number of pseudogenes was calculated using *w*Mel as a reference genome as it had the highest completeness score among strains (CheckM 99.79%, BUSCO 99.5%). Pseudogene indicators were classified as frameshifts, missing stop codons, and internal stop codons.

### Phylogenomic analysis

The core gene alignment file (core_gene_alignment.aln) from Roary was converted to phylip format for phylogenomic analysis with PhyloBayes MPI, which uses the CAT-GTR model to predict long-branch interactions (Lartillot et al., 2013). Two independent chains were run parallelly, and the consensus was obtained by pooling all the trees of the independent chains using the bpcomp package in PhyloBayes MPI. Using the burn-in of 1,000, and sub-sampling every 10 trees, the bpcomp program calculated the largest (maxdiff) and mean (meandiff) discrepancy observed across all bipartitions. The core gene alignment file (core_gene_alignment.aln) from Roary was also imported to the Geneious Prime software for phylogeny construction with MrBayes (Ronquist et al., 2012). For MrBayes, posterior probabilities were reported for supported nodes from Bayesian 50% majority rule, with the GTR+G model with four rate categories. Additional phylogenomic comparisons were performed with RAxML using the GTR+Gamma model with support shown for 100 bootstrap replicates (Stamatakis, 2014). To test the effects of fragmented sequences and sequences with long branches, additional analyses were performed with these sequences removed.

Phylogenetic analysis of 16S rRNA gene sequences from different *Wolbachia* strains and out-groups was performed in RAxML using the GTR + Gamma model with support from 100 bootstrap replicates (Stamatakis, 2014). In addition, based on previous PCR assays that screened *Wolbachia* from different nematode populations, a *Wolbachia* 16S rRNA gene from *Helicotylenchus* sp. from Florida (Kaur and Brown, 2024) is also included in the phylogenetic analysis. A further 16S rRNA gene was included from a separate project in our laboratory (unpublished) based on an assembly of reads from *Heterodera sojae* from soybean roots from South Korea (SRR25626476). Phylogenetic analysis of COI genes from *Heterodera* spp., including COI genes recovered from Nanopore-assembled *H. schachtii* IRS assembly, was also performed in RAxML using the GTR + Gamma model with support from 100 bootstrap replicates (Stamatakis, 2014).

### Comparison with RNA-seq datasets

Based on additional blastn screens of transcriptomic (RNA-seq) SRA datasets from *H. schachtii* in NCBI, several additional samples with *Wolbachia*-like matches were analyzed. For these samples, Illumina reads were downloaded and *de novo* assembled using rnaSPAdes (Bushmanova et al., 2019), and assembled transcripts matching the *Wolbachia* 16S rRNA gene were aligned and analyzed as described above for phylogenetic analysis.

### Gene ontology enrichment analysis

Gene ontology enrichment analysis was performed to interpret the enriched GO terms for new *Wolbachia* strain against different *Wolbachia* species from supergroups A, B, C, D, E, F, J, M, S, and T and supergroup L. Functional GO enrichment was assessed using topGO v2.4.0 (Alexa and Rahnenfuhrer, 2024) which evaluates GO term graph topology and uses the ‘weight01.fisher’ algorithm to create test statistics, returning corrected *p*-values not affected by multiple testing. TopGO was performed in R using the script aip_topgo_usage.consider_universe.R^3^ for multiple gene subsets depicted in Venn diagrams using the ‘diff’ and ‘universe’ sets of genes. GO-figure was used to visualize gene ontology enrichment, summarizing the list of GO terms based on their semantic similarity and producing scatterplots with clustered GO terms of similar functions (Reijnders et al., 2021).

## Results

### *Wolbachia* found in a *Heterodera schachtii* but absent in *Meloidogyne* spp., *Globodera* spp., and other *Heterodera* spp.

**Figure 1:**
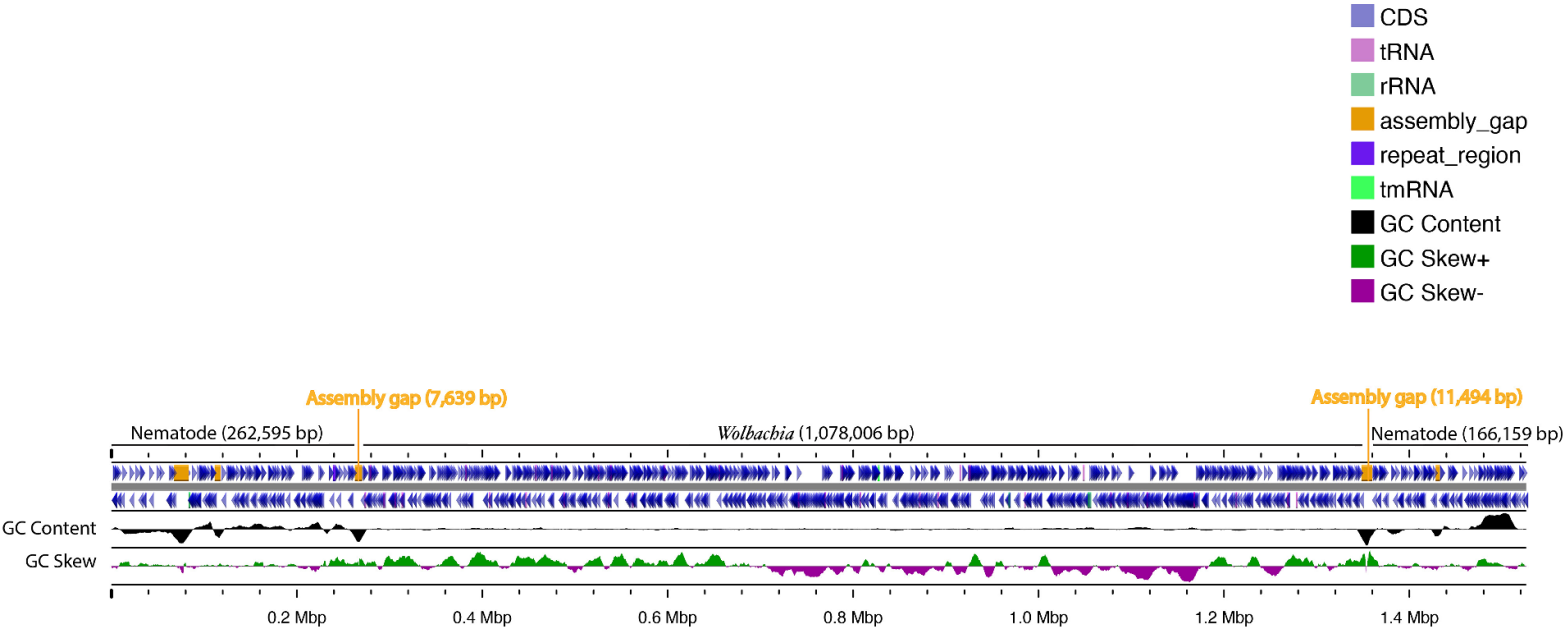
Representation of the *Heterodera schachtii* isolate IRS assembly 1.5 Mbp scaffold (JAIZDD010000066.1) harboring a *Wolbachia*-like sequence (1.1 Mbp) flanked by assembly gaps (Ns) and nematode-matching regions.

**Figure 2:**
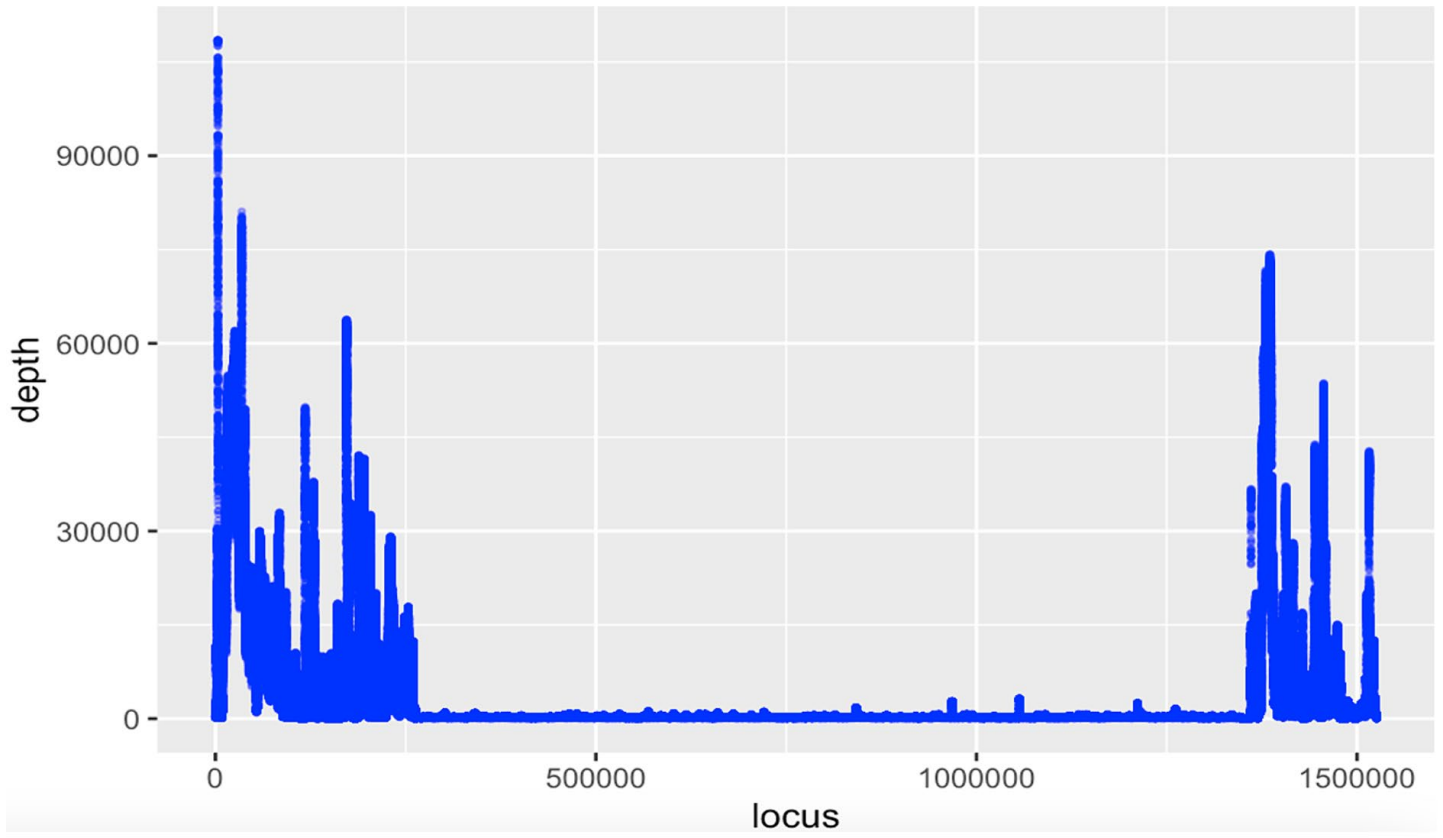
Coverage plot of the 1.5 Mbp scaffold (JAIZDD010000066.1 from NCBI project accession PRJNA767548) for *Heterodera schachtii* isolate IRS harboring a *Wolbachia*-like sequence (1.1 Mbp) flanked by assembly gaps (Ns) and nematode-matching regions. Mapped nanopore reads were derived from the same project (SRR16146220-SRR16146534 and SRR16675965-SRR16675966 and SRR28675229-SRR28675543).

No *Wolbachia* was detected in DNA read (SRA) data and WGS assemblies for *Meloidogyne* spp. and *Globodera* spp. (Supplementary Tables 1, 2), but among the seven *Heterodera* spp. WGS assemblies, one assembly from *Heterodera schachtii* (an isolate named ‘IRS’ from the Netherlands, sequenced using Oxford Nanopore Technologies, with NCBI accession GCA_020449115.1) was positive for *Wolbachia* (Table 2). Analysis of this assembly (previously assembled using Wtdgb2 v.2.3) revealed a large (1.5 Mbp) scaffold (JAIZDD010000066.1) containing a large *Wolbachia* region (1.1 Mbp) between scaffold positions 270,235 and 1,348,240 flanked by nematode genes. The scaffold’s upstream flanking region matched nematodes, based on blastn, and was 0.16 Mbp. The downstream flanking region also matched nematodes and was 0.25 Mbp (Figure 1). Based on the mapping of the mapped Nanopore reads from the same sample (SRA accessions SRR16146220-SRR16146534 and SRR16675965-SRR16675966 and SRR28675229-SRR28675543 from the same project) to this scaffold, the flanking nematode regions had an average fold coverage of 15,244X (upstream) and 18,099X (downstream), whereas the *Wolbachia-matching region* had an average fold coverage of 829X (Figure 2).

**Table 2:** Whole-genome sequencing (WGS) assemblies available for *Heterodera* spp. in NCBI with accompanying DNA reads’ SRA accessions.

*Heterodera* spp.	GenBank accession	Submitter	WGS project and SRA accessions	Sequencing platform	Total sequence length (Mb)	Number of scaffolds	Presence of *Wolbachia*
*Heterodera schachtii* isolate IRS	GCA_020449115.1	Wageningen University and Research, Netherlands	JAIZDD01 SRR16146220 to SRR16146534, SRR16675965 to SRR16675966, SRR28675229 to SRR28675543	Nanopore	190	705	Yes
*Heterodera schachtii* isolate Bonn	GCA_019095935.1	The *H. schachtii* genome sequencing consortium, Germany	JAHGVF01 SRR15101032, SRR15496954, SRR15603410 to SRR15603442	PacBio	179	395	No
*Heterodera schachtii* isolate Bonn	GCA_023374025.1	The *H. schachtii* genome sequencing consortium, Germany	SIJG01 (no SRA)	PacBio	179	395	No
*Heterodera schachtii* isolate Gr-Nem-00856	GCA_034696305.1	LOEWE Centre for Translational Biodiversity Genomics, Germany	JAQFZV01 SRR21208377	Illumina	190.8	194,125	No
*Heterodera glycines* strain OP25	GCA_000150805.1	Monsanto, USA	ABLA01 (no SRA)	PacBio	82	34,708	No
*Heterodera glycines* strain X12	GCA_015680885.1	Institute of Industrial Crops	VAPQ01 SRR9644798 to SRR9644808	PacBio	141	257	No
*Heterodera carotae* isolate Calama	GCA_024500135.1	USDA-ARS	JAKKIK01 SRR16603784	Illumina	95	17,845	No

Based on a blastn search against a custom nematode COI gene database, this Nanopore assembly for *H. schachtii* isolate IRS had two COI genes present in the assembly; one was most similar to *H. medicaginis* (382 bp hit with 90.576 percent identity to the COI gene), and other was *H. schachtii* (382 bp hit with 99.738 percent identity to the COI gene). The *H. medicaginis*-like COI region had an average fold coverage of 55,196X, whereas the *H. schachtii*-like COI region had an average fold coverage of 11,052X.

**Figure 3:**
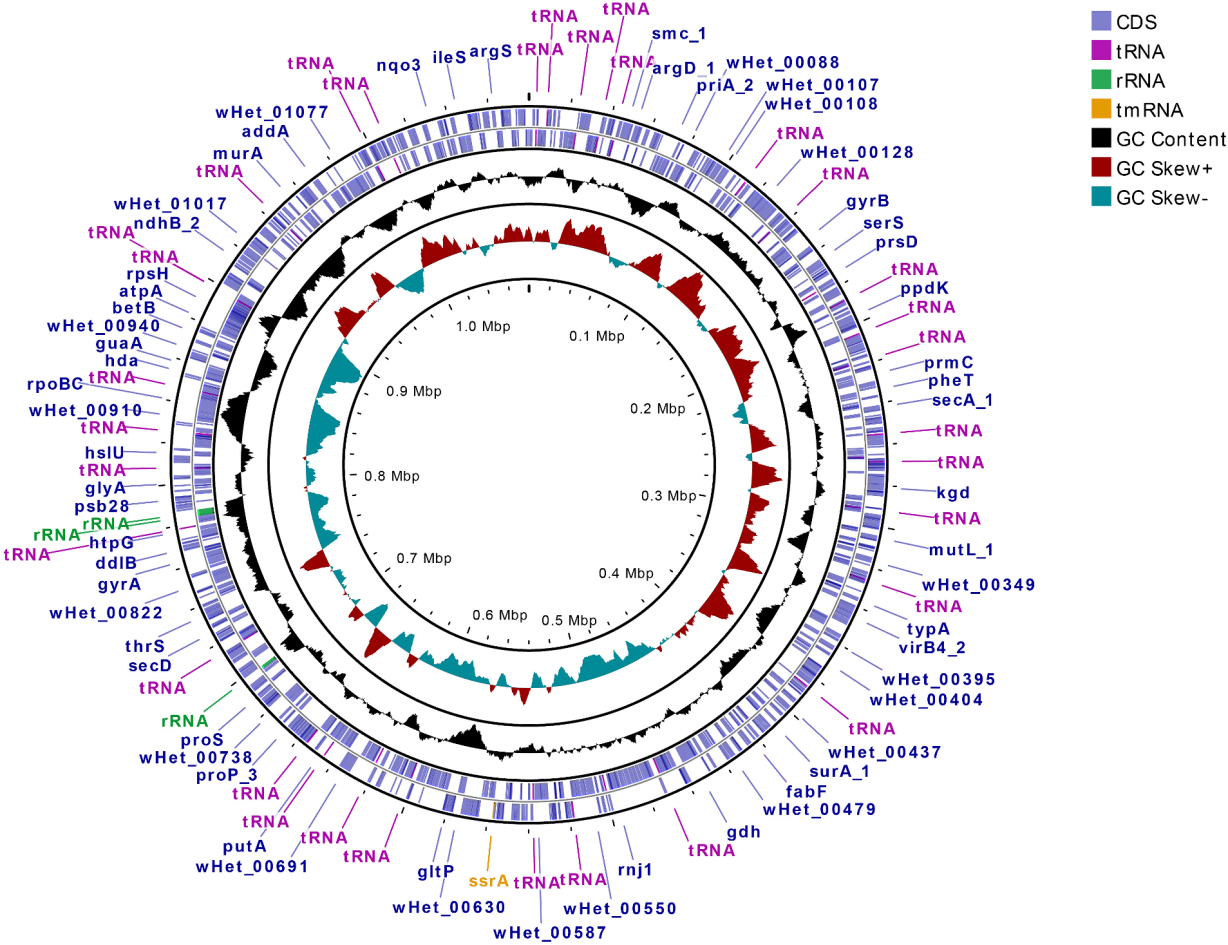
Circular genome representation of new *Wolbachia* strain *w*Het derived from re-assembly and circularization of overlapping scaffold ends, showing CDS, tRNA, rRNA, tmRNA, GC content, and GC skewness. Outermost circle represents the position of coding sequences (CDS) and RNA genes on the forward and reverse strand. Inner circles display plots of GC content and GC skew, respectively, showing their deviation from the average for the entire sequence.

Upon visualization of the *Wolbachia-*containing scaffold (JAIZDD010000066.1) in Geneious Prime, the *Wolbachia-*like region was flanked by a number of non-called bases (Ns) in upstream and downstream regions in the connecting junctions between *Wolbachia-*like and nematode regions (Figure 1). However, mapping and re-assembly of SRA raw reads of this WGS project to this *Wolbachia-*containing scaffold revealed an additional 1,802 bp region downstream of this *Wolbachia-*like region that extended the main scaffold sequence across the downstream Ns in the assembly (i.e., filling the gap) and formed a 262 bp overlap with forward end of the main scaffold at the upstream junction with Ns, such that assembling the 1,802 bp region and the original scaffold created a complete *Wolbachia* circular genome (Figure 3) (denoted *w*Het, hereafter). Quast assembly statistics revealed this 1,079,546 bp (1.1 Mbp) sequence as a genome in single contig with GC content 32.59%, which is in the range of other *Wolbachia* strains (Supplementary Table 3).

### Phylogenetic analysis of *w*Het with other *Wolbachia* strains and out-groups

**Figure 4:**
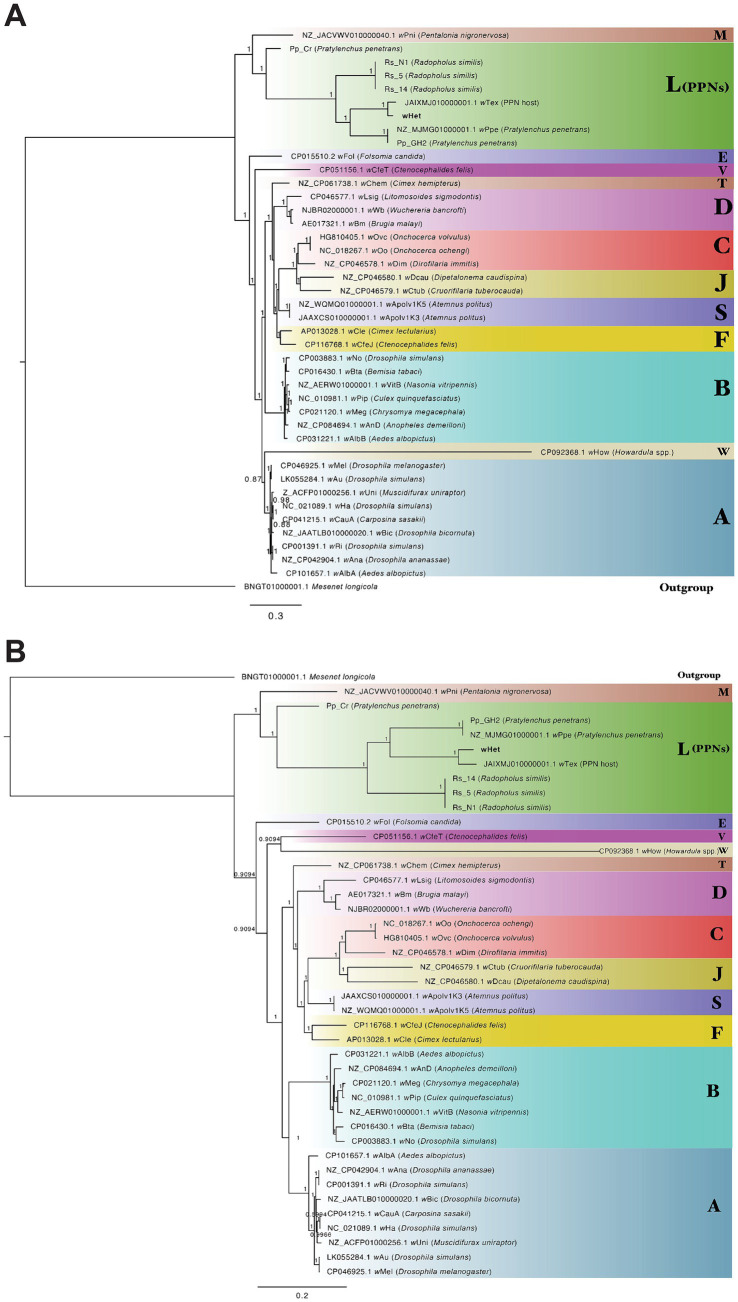
Phylogenetic trees of *Wolbachia* strains and out-group generated from an alignment of 37 core genes (32,748 nucleotide positions) **(A)** analyzed with PhyloBayes using the CAT-GTR model with posterior probabilities shown on branches **(B)** analyzed with MrBayes using the CAT-GTR model with posterior probabilities shown on branches. Strain *w*Het is shown in bold. Letters represent supergroups A (insects), B (insects), C (filarial nematodes), D (filarial nematodes), F, J, M, S, T, V, and W and supergroup L (plant-parasitic nematodes).

Ortholog analysis of the novel *Wolbachia* strain (*w*Het) against published *Wolbachia* genomes from supergroups A (insects), B (insects), C (filarial nematodes), D (filarial nematodes), E, F, J, M, S, T, V, and W and supergroup L (plant-parasitic nematodes) and out-group *Ca.* Mesenet longicola revealed 37 core genes (orthologs) shared across all the genomes. Phylogenetic analysis of the 37-gene block (32,748 nucleotide positions) with the PhyloBayes package produced bpcomp results with the largest discrepancy results of maxdiff = 0.0414118 across all bipartitions, which indicated a good run (if maxdiff < 0.1) for independent chain runs. The consensus tree obtained using PhyloBayes MPI (Figure 4A) and MrBayes (Figure 4B) confirmed that this novel *Wolbachia* genome belonged to supergroup L, comprising *Wolbachia* from PPNs. The closest relative to *w*Het was *w*Tex, followed by *w*Ppe and Pp_GH2, with evidence supporting for the L monophyly and relationships in this clade. Pp_Cr was highly divergent from *P. penetrans Wolbachia* strains as its assembly was highly fragmented with a coding density of 57.47% (Table 3). *Wolbachia* strains from insects were grouped together for supergroup A and supergroup B. *Wolbachia* strains from filarial nematodes, supergroup C, and supergroup D formed separate groups (Figure 4). The results for all groups for the full alignment, and additional alignments with gaps removed, fragmented sequences removed, or long-branch sequences (*w*How) removed, and analyses using RAxML produced identical phylogenies with similar node support in all cases (Supplementary Figure 1), except for the relative position of the long branch *w*How. Strain *w*How grouped as sister to supergroup A in PhyloBayes CAT analysis (Figure 4A), as sister to supergroup V (*w*CfeT) in MrBayes analysis (Figure 4B), and as sister to supergroup L with RAxML analysis.

**Table 3:** Genomic features of *w*Het compared to closely related *Wolbachia* strains and *w*Mel (which was included in the comparison as it was used as reference to calculate the pseudogenes).

Genomic features/strain	*w*Het	*w*Ppe	*w*Tex	Rs_N1	Rs_5	Rs_14	Pp_GH2	Pp_Cr	*w*Mel
Host organism	*H. schachtii*	*P. penetrans*	PPN (predicted *Helicotylenchus* sp.)	*R. similis*	*R. similis*	*R. similis*	*P. penetrans*	*P. penetrans*	*D. melanogaster*
Genome size (Mb)	1.1 Mb	0.97 Mb	1.01 Mb	0.95 Mb	0.92 Mb	0.95 Mb	1.03 Mb	0.97 Mb	1.3 Mb
Non-hypothetical proteins/hypothetical proteins	683/506	637/364	632/394	623/303	613/287	660/292	544/394	640/380	785/522
tRNA genes	35	35	37	42	40	57	37	43	34
rRNA genes	3	3	3	7	4	18	9	9	3
%GC	32.59	32.16	33.49	33.31	32.99	32.82	32.56	32.38	35.23
% completeness (CheckM)	96.05	97.85	82.79	97.84	97.19	97.42	99.15	68.82	99.79
% completeness (BUSCO)	88.5	95.3	83.3	97.8	97.3	96.9	98.3	69.2	99.5
pseudogenes	2	13	9	12	5	3	2	6	0
#frameshifts	1	8	1	9	0	0	0	1	0
#missing start codon	0	0	0	0	0	0	0	0	0
#missing stop codon	1	0	3	3	5	3	0	3	0
#internal stop codon	0	5	5	0	0	0	2	2	0
Ankyrin genes	3	0	25	0	0	0	0	0	0
Transposases	10	0	34	5	3	3	3	3	75
Phage-related genes	0	0	0	0	0	0	0	0	2
Coding density %	86.13	87.64	78.14	83.55	84.18	84.50	83.23	57.47	86.16

**Figure 5:**
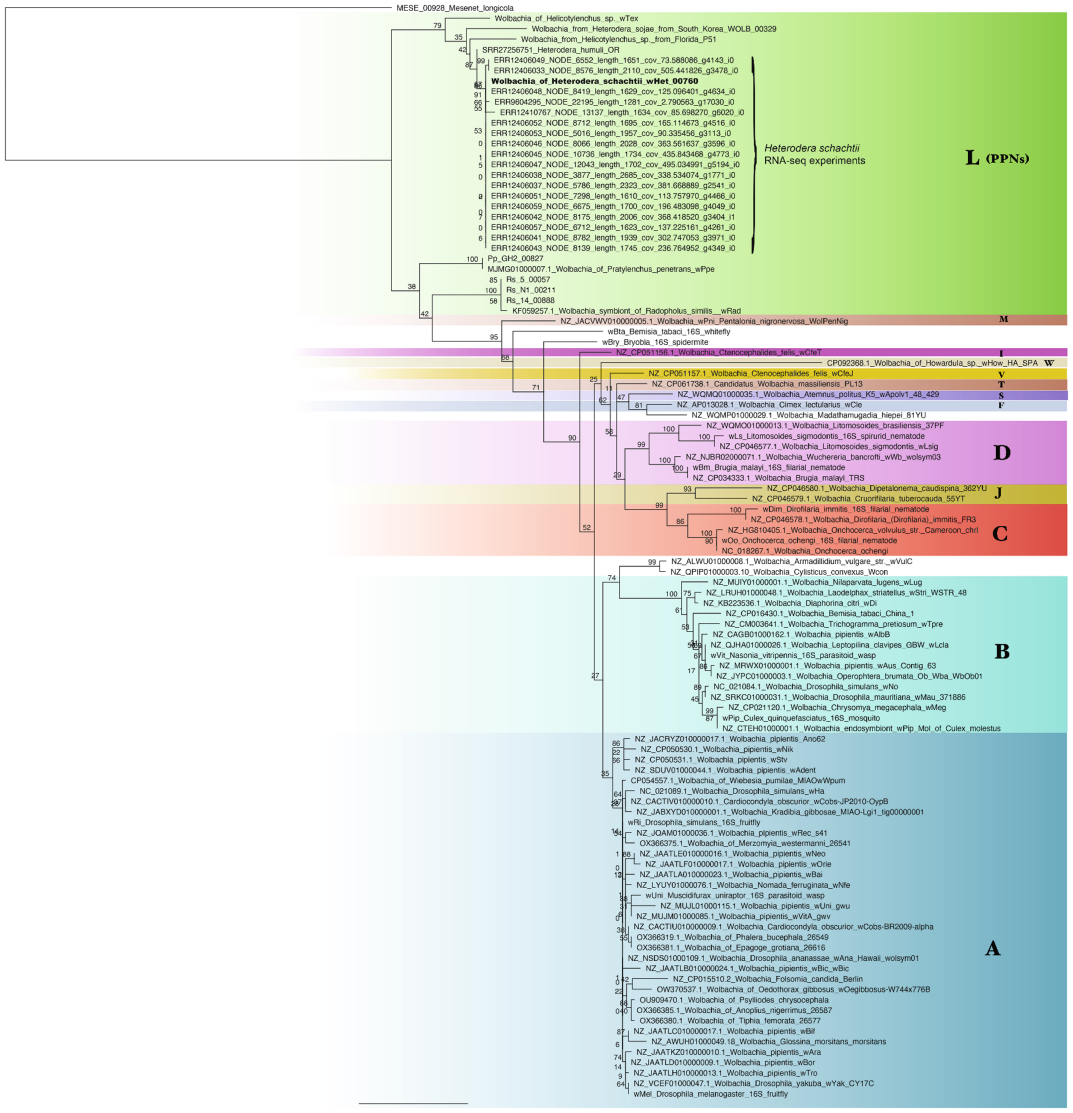
Phylogenetic tree of *Wolbachia* strains and out-groups generated by RAxML with the GTR + Gamma model from a 1,530 bp alignment of the 16S rRNA gene, with support from 100 bootstrap replicates shown on branches. The clade including *w*Het is shown in bold. Samples from SRA (SRR/ERR) datasets were assembled from publicly available reads on NCBI. Letters represent supergroups A (insects), B (insects), C (filarial nematodes), D (filarial nematodes), F, M, S, and T and supergroup L (plant-parasitic nematodes).

Phylogenetic analysis of the 16S rRNA gene performed in RAxML using the GTR + Gamma model and in MrBayes revealed similar results to the core gene phylogeny, but here, *w*Het was shown also to be closely related to a *Wolbachia* strain isolated from *H. sojae* (soybean) roots from South Korea and a *Wolbachia* strain isolated from a *Helicotylenchus* sp. from Florida. Strain wHet was also closely related to an isolate of *Heterodera humuli* from Oregon (reads assembled from SRR27256751), and *w*Tex. Evidence supported the monophyly and relationships in this clade (Figure 5; Supplementary Figures 2, 3), regardless of phylogenetic method or choice of out-groups. In addition, two transcriptomic datasets from *H. schachtii* RNA-seq projects on NCBI generated close matches to the 16S rRNA gene sequence of *w*Het (project PRJEB71637 from University of Cambridge, and a project examining roots of *Arabidopsis thaliana* infected with *H. schachtii* from Wageningen University with sample accession SAMEA14093318) (Figure 5; Supplementary Figure 2).

Phylogenetic analysis of the COI gene from *Heterodera* spp. including two COI genes recovered from the *H. schachtii* isolate IRS assembly showed that *H. medicaginis* COI and *H. schachtii* COI from this assembly were divergent in the tree (Supplementary Figure 4).

### Comparative genome features of *w*Het against other *Wolbachia* strains

The final predicted circular genome from *w*Het had 96.05% estimated completeness (based on the marker gene sets present/absent that are specific to a genome’s inferred lineage within a reference genome tree in reference databases in checkM), 88.5% estimated completeness (based on BUSCO analysis), 683 predicted proteins with known functions, 506 predicted proteins with unknown functions, 35 tRNA genes, three rRNA genes, three ankyrin region containing genes, and 10 transposases (Table 3). All the PPN supergroup *Wolbachia* strains (*w*Het, *w*Ppe, *w*Tex, Rs_N1, Rs_14, Rs_5, Pp_Cr, and Pp_GH2) had no predicted prophage-related genes, unlike *Wolbachia* strains from other clades except for supergroups C and J. Compared to *w*Mel, *w*Het had two pseudogenes, one frameshift, and one gene with a missing stop codon. Further examination revealed that *w*Het, such as *w*Ppe, *w*Tex, Rs_N1, Rs_14, Rs_5, Pp_Cr, and Pp_GH2, lacked homologs to known CI (cytoplasmic incompatibility) associated genes, namely, *cifA* and *cifB*, plasmid-associated genes, and WO prophages. *w*Het also had *thiE* (encoding thiamine phosphate synthase), such as others in the L (PPN) supergroup except for *w*Tex. Various genes related to heme pathways were found in *w*Het, for example, *hemA* (encoding 5-aminolevulinate synthase), *hemB* (encoding delta-aminolevulinic acid dehydratase), *hemC* (encoding porphobilinogen deaminase), *hemE* (encoding uroporphyrinogen decarboxylase), *hemF* (encoding oxygen-dependent coproporphyrinogen-III oxidase), *hemH* (a ferrochelatase), *ctaA* (encoding heme A synthase), and *ctaB* (encoding protoheme IX farnesyltransferase), such as all others in L supergroup, except for *hemH* missing in *w*Tex, *ctaB* missing in Rs_14, and *catA* and *hemE* missing in Pp_Cr.

### Orthologs among *w*Het and related *Wolbachia* strains

Although *w*Het was closest to *w*Tex in phylogenomic analyses, genome content similarity among this strain and others was less clear, likely due to the incompleteness of several other PPN *Wolbachia* genomes. Orthologous gene cluster comparisons showed *w*Het shared the most genes with Pp_GH2, Rs_5, and Rs_14 (549 genes shared with each) and similarly high numbers of genes with Rs_N1 and *w*Ppe (547 and 546, respectively). *w*Het and *w*Tex shared 429 genes, with 110 genes unique to *w*Het and 32 genes unique to *w*Tex, likely a lower number because the *w*Tex genome was incomplete (Supplementary Figure 5). Another *Wolbachia*, *w*How, from an entomoparasitic tylenchid nematode and having a highly reduced genome, shared 401 genes with *w*Het, with 209 genes unique to *w*Het and 25 genes unique to *w*How.

Across members of supergroup L, combined analysis of *Wolbachia* strains from *Pratylenchus penetrans* as well as *w*Tex (unknown host) showed 365 genes shared with *w*Het and 32 genes unique to *w*Het, whereas *Wolbachia* strains from *Radopholus similis* shared 546 genes with *w*Het, 60 genes being unique to *w*Het (Supplementary Figure 6). Supergroup C *Wolbachia* strains *w*Oo (*Wolbachia* endosymbiont of *Onchocerca ochengi*), *w*Ovc (*Wolbachia* endosymbiont of *Onchocerca volvulus*), and *w*Dim (*Wolbachia* endosymbiont of *Dirofilaria immitis*) shared 484 genes with *w*Het, with 97 unique genes in *w*Het. Supergroup D *Wolbachia* strains, *w*Bm (*Wolbachia* endosymbiont of *Brugia malayi*), *w*Lsig (*Wolbachia* endosymbiont of *Litomosoides sigmodontis*), and *w*Wb (*Wolbachia* endosymbiont of *Wuchereria bancrofti*) shared 506 genes with *w*Het. Supergroup J *Wolbachia* strains *w*Dcau (*Wolbachia* endosymbiont of *Dipetalonema caudispina*) and *w*Ctub (*Wolbachia* endosymbiont of *Cruorifilaria tuberocauda*) shared 473 genes with *w*Het. Another close relative *w*Pni (*Wolbachia* endosymbiont of *Pentalonia nigronervosa*) shared 538 genes with *w*Het (Supplementary Figure 6).

### Genome length versus GC content comparing *w*Het to other *Wolbachia* strains

**Figure 6:**
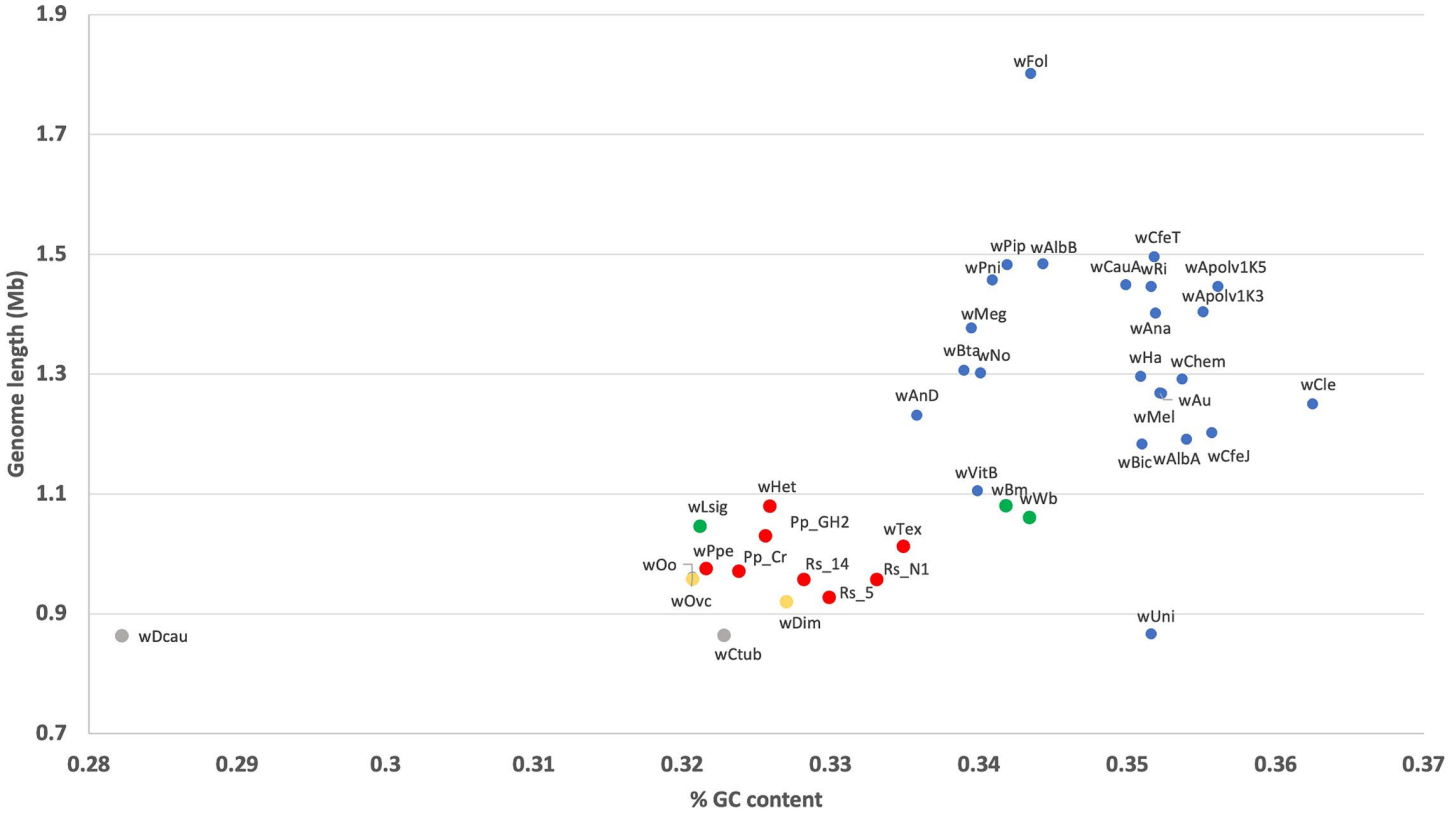
GC content versus genome length for different *Wolbachia* strains including the new *Wolbachia* strain *w*Het. Red color represents supergroup L (PPNs), yellow color represents supergroup C (filarial nematodes), green color represents supergroup D (filarial nematodes), and blue color represents supergroups A and B (insects).

Comparing assembly length versus GC content showed that *w*Het followed the trend for all *Wolbachia* strains in which lower GC content is associated with smaller genomes (Figure 6). Strain *w*Het had a genome size and GC content similar to that of other supergroup L members, which was closer to that of supergroups D and C than to strains from groups A and B.

### Gene ontology enrichment analysis

GO-figure plots showed significant differences in enriched GO terms in *w*Het compared to relatives and out-groups (Supplementary Figure 7). These plots depict GO term semantic similarity in predicted gene products, where the size of the node reflects the number of genes annotated to that term. *w*Het enriched GO terms showed more overlapping semantic space with supergroups C, D, and L, as compared to supergroups A and B, depicting similar functional relatedness in biological processes with nematode *Wolbachia* supergroups. However, there were significant overlaps with supergroup S. When *w*Het was compared individually to supergroup L *Wolbachia* strains from *P. penetrans*, *R. similis*, and *w*Tex, *w*Tex shared the most semantic space overlap with *w*Het (Supplementary Figure 7).

From topGO analysis, *w*Het did not show any significant differences compared to *w*Tex and supergroup B *Wolbachia* strains in significantly enriched GO terms, but as compared to *Wolbachia* strains from other supergroups (A, B, C, D, F, S, T, and L), a few significant differences were observed. The results showed that *w*Het was enriched for nitrogen metabolic processes compared to supergroups C, D, J, S, and T, as well as lipid catabolic processes compared to supergroup T (Table 4).

**Table 4:** Significantly enriched gene ontology (GO) categories based on topGO analysis for the pangenome of *Wolbachia w*Het compared to the pangenome *Wolbachia* strains from different supergroups, including genes shared between pangenomes.

GO.ID	Term	Annotated	Significant	Expected	*p*-value
*w*Het vs. Supergroup A					
Biological processes					
–	–	–	–	–	–
Cellular components					
–	–	–	–	–	-
Molecular functions					
GO:0042802	Identical protein binding	16	4	0.8	0.0062
*w*Het vs. Supergroup C					
Biological processes					
GO:0006807	Nitrogen compound metabolic process	325	45	51.66	0.042
Cellular components					
GO:0016020	Membrane	164	31	22.08	0.0098
GO:0016021	Integral component of membrane	7	3	0.94	0.0481
Molecular functions					
GO:0042802	Identical protein binding	17	8	1.98	0.00095
GO:0003677	DNA binding	62	14	7.23	0.02985
*w*Het vs. Supergroup D					
Biological processes					
GO:0006807	Nitrogen compound metabolic process	345	25	29.5	0.013
Cellular components					
–	–	–	–	–	–
Molecular functions					
GO:0042802	Identical protein binding	24	6	1.8	0.0043
*w*Het vs. Supergroup F					
Biological processes					
GO:0051289	Protein homotetramerization	5	2	0.36	0.0429
Cellular components					
–	–	–	–	–	–
Molecular functions					
GO:0042802	Identical protein binding	34	7	2.22	0.0061
*w*Het vs. Supergroup J					
Biological processes					
GO:0051301	Cell division	23	7	3.31	0.0314
GO:0006807	Nitrogen compound metabolic process	322	36	46.28	0.0455
Cellular components					
GO:0016020	Membrane	156	36	23.07	0.00026
GO:0005829	Cytosol	26	8	3.84	0.2521
Molecular functions					
GO:0042802	Identical protein binding	17	8	2.2	0.0017
*w*Het vs. Supergroup S					
Biological processes					
GO:0006807	Nitrogen compound metabolic process	374	24	30.15	0.0135
Cellular components					
–	–	–	–	–	–
Molecular functions					
GO:0042802	Identical protein binding	31	7	2.13	0.0142
GO:0008324	Cation transmembrane transporter activity	28	6	1.92	0.0365
*w*Het vs. Supergroup T					
Biological processes					
GO:0006807	Nitrogen compound metabolic process	342	27	31.8	0.0159
GO:0016042	Lipid catabolic process	5	3	0.46	0.0435
Cellular components					
GO:0005829	Cytosol	33	6	2.79	0.048
Molecular functions					
GO:0042802	Identical protein binding	25	6	2.1	0.0424
*w*Het vs. supergroup L					
Biological processes					
–	–	–	–	–	–
Cellular components					
GO:0005829	Cytosol	36	4	1.36	0.039
Molecular functions					
GO:0042803	Protein homodimerization activity	11	2	0.33	0.039

Compared to supergroup L strains, *w*Het had significantly enriched GO terms for cytosol (cellular component) and protein homodimerization activity (molecular function) against *Wolbachia* strains from *P. penetrans* and significantly enriched GO terms for regulation of cell shape (biological process) and protein homodimerization activity (molecular function), against *Wolbachia* strains from *R. similis* (Table 5). There were similar differences as compared to supergroup A and F strains (Table 4). There were also similar differences observed as compared to supergroup D and supergroup S strains, where *w*Het had enriched GO terms for nitrogen compound metabolic process (biological process), identical protein binding (molecular function) against both supergroups, and enriched cation transmembrane transporter activity (molecular function) specifically against supergroup S (Table 4).

**Table 5:** Significantly enriched gene ontology (GO) categories based on topGO analysis for the pangenome of *Wolbachia w*Het compared to the pangenome of plant-parasitic nematode-associated *Wolbachia* (supergroup L), including genes shared between pangenomes.

GO.ID	Term	Annotated	Significant	Expected	*p*-value
*w*Het vs. *P. penetrans Wolbachia* strains					
Biological processes					
–	–	–	–	–	–
Cellular components					
GO:0005829	Cytosol	31	7	1.89	0.0013
Molecular functions					
GO:0042803	Protein homodimerization activity	9	3	0.46	0.0082
*w*Het vs. *R. similis Wolbachia* strains					
Biological processes					
GO:0008360	Regulation of cell shape	17	5	1.22	0.0048
Cellular components					
–	–	–	–	–	–
Molecular functions					
GO:0042803	Protein homodimerization activity	5	3	0.37	0.0035

## Discussion

Despite *Wolbachia*’s widespread distribution and biological importance in arthropods and filarial nematodes, its distribution and role in plant-parasitic nematodes (PPNs) are inadequately known. Uncovering the potential roles of PPN *Wolbachia* is of great interest to the agricultural sector as these endosymbionts could be explored as possible candidates for developing biocontrol measures to reduce PPNs. This study sought to find PPN *Wolbachia* from public databases and resulted in the discovery of a new hidden *Wolbachia* strain, designated *w*Het, from long-read sequences from NCBI’s SRA database from a *Heterodera schachtii* assembly from the Netherlands. Previous studies have reported the occurrence of *Wolbachia* in PPNs across South America, North America, Africa, and Asia (Haegeman et al., 2009; Wasala et al., 2019; Wasala et al., 2023), but this is the first genome study demonstrating the occurrence of *Wolbachia* from a species of *Heterodera* in Europe.

Initially, it appeared that this new PPN *Wolbachia* strain, *w*Het, might be a large putative HGT (horizontal gene transfer) from *Wolbachia* to the host, perhaps similar to the whole-genome *Wolbachia* HGT found in *Drosophila ananassae* (Hotopp et al., 2007), but our analyses suggest this is not an HGT but instead represents a *Wolbachia* symbiont. In support of this argument, we discuss four types of evidence. First, the presence of long string of Ns directly flanking both ends of the *Wolbachia* region with few to no Ns in the *Wolbachia* region itself or in the flanking nematode regions suggests an assembly artifact. Second, our analyses showed a substantial difference in the average fold coverage of the *Wolbachia-*like region and flanking nematode-like regions, which suggest, again, that the apparent integration of the *Wolbachia* region is an assembly artifact. Third, our mapping and assembly that recovered the downstream (1,802 bp) end of the *Wolbachia* genome from SRA reads clearly gap-filled the Ns flanking the *Wolbachia* scaffold and uncovered a significant overlap (262 bp) between the upstream and downstream ends of the new combined assembly, to form a complete circular genome assembly. Finally, additional analysis of other *H. schachtii* projects based on RNA, rather than DNA, revealed a high-coverage 16S rRNA sequence identical with that of *w*Het suggesting the symbiont itself expresses its ribosomal RNA.

While our genomic analyses suggested the absence of *Wolbachia* in all other *H. schachtii* DNA assemblies from Germany (Collins et al., 2023; Siddique et al., 2022), we did detect *Wolbachia w*Het in other *H. schachtii* SRA data from transcriptome projects. Specifically, our analysis of *H. schachtii* RNA transcriptome assemblies revealed two separate sequencing projects with clear matches to *w*Het, supporting the hypothesis that the WGS-recovered *Wolbachia* sequence from the Nanopore ‘IRS’ project likely represents a real *Wolbachia* symbiont rather than a nematode host genome integration, as discussed above. Particularly, the discovery of high-coverage 16S rRNA sequences matching *w*Het from these transcriptome projects would be unexpected from a mere genome integration. Instead, recovery of *Wolbachia* 16S rRNA from these projects which used polydT library selection is a strong indicator that the ribosomal RNA itself is expressed and abundant enough to ‘contaminate’ the mature mRNA pulled down in the library preparation. To our knowledge, there is no study demonstrating that eukaryote nuclear-encoded *Wolbachia* rRNA genes would be expressed effectively enough to produce this result. Nevertheless, the apparent absence of *Wolbachia* from some DNA and RNA experiments would appear to indicate a result of biological importance: *w*Het may have variable presence or absence across isolates of this host. This pattern is consistent with that of another PPN *Wolbachia*, *w*Ppe (Wasala et al., 2019), possibly indicating the strain has a facultative, rather than obligate role.

Steps to determine the host of *w*Het in this assembly revealed the presence of another nematode COI gene in the *H. schachtii* (sugarbeet nematode) assembly from the Netherlands, which was related at ~90% sequence identity to *H. medicaginis* (alfalfa cyst nematode), suggesting that this recovered sequence might reflect a cryptic and/or unnamed species present in the sample along with true *H. schachtii*. We note that our analyses suggest that NCBI records for *H. medicaginis* COI genes may reflect taxonomic issues or a polyphyletic species (see Supplementary Figure 2). To date, no SRA data were available on NCBI for *H. medicaginis* to search for *Wolbachia* in this nematode species. However, since no other species contamination was detected except for these two *Heterodera* species, the formation of a long branch in the PPN *Wolbachia* supergroup from tylenchids for this symbiont suggests a longstanding relationship of *Wolbachia* within this *H. schachtii*-like host clade, adding Heteroderidae to the short list of families of tylenchid nematode hosting *Wolbachia* along with Pratylenchidae (Brown, 2018; Brown et al., 2016; Haegeman et al., 2009; Kaur and Brown, 2024).

Our assembled *w*Het genome of 1.1 Mbp represents the most complete PPN *Wolbachia* discovered to date: Other PPN *Wolbachia* draft genomes (*w*Ppe, *w*Tex, Pp_Cr, Pp_GH2, Rs_14, Rs_5, and Rs_N1) are fragmented. Hence, this *w*Het genome provides a useful resource for improved genomic analyses. Genomic features and phylogenetic analysis of *w*Het compared to other *Wolbachia* strains confirmed that this strain belongs to supergroup L, a PPN-type *Wolbachia* clade. Supergroup L is a monophyletic supergroup in the *Wolbachia* phylogeny, including *w*Tex, *w*Ppe, Rs_N1, Rs_14, Rs_5, Pp_Cr, Pp_GH2, and *w*Rad, and most analyses placed *w*Het and group L closet to the nearest out-groups of genus *Wolbachia* (i.e., *Ca.* Mesenet longicola and other Anaplasmataceae). Despite consistency of phylogenetic results for most *Wolbachia* in this study, the long branch of *w*How changed position depending on the phylogenetic method used. Although it was not the focus of this study, the varying position of *w*How here, and compared to a previous study (Dudzic et al., 2022), may reflect long-branch attraction (LBA) effects. Notably, the latter study using the maximum likelihood-based program IQ-TREE was more similar to our RAxML results, whereas our PhyloBayes MPI (CAT-GTR model) which reportedly corrects for some LBA (Lartillot et al., 2013) placed *w*How further from out-groups. We suggest further taxonomic sampling of W and L supergroups and refinement of the phylogenetic analysis models and methods will be required to overcome possible long-branch attraction artifacts.

Our phylogenetic analysis consistently placed *w*Het closer to *w*Tex than to other *Wolbachia* strains, whereas its gene repertoire was closer to Pp_GH2, Rs_5, and Rs_14. This gene repertoire result may be due to an artifact of the incompleteness of the *w*Tex draft assembly. We also found the PPN *Wolbachia* as a group (including *w*Het) had a core gene repertoire more similar to that of *w*Pni (*Wolbachia* endosymbiont of banana aphid, *Pentalonia nigronervosa*) than to other *Wolbachia* characterized thus far. This finding is consistent with previous analyses (Weyandt et al., 2022), suggesting evolutionary relatedness and potentially similar biological processes of the host, such as plant feeding. In terms of gene sharing with supergroup L, *w*Het had 32 unique genes as compared to *Wolbachia* strains from *P. penetrans* and 60 unique genes as compared to *Wolbachia* strains from *R. similis*, suggesting that these genes may be strain-specific or play a role in the biology of each strain. However, most of these accessory genes were annotated as ‘hypothetical protein,’ as for other *Wolbachia* strains, making it difficult to infer functional differences.

From analysis of the remaining *w*Het genes that could be annotated to function, it appeared that this strain was very similar to *w*Tex, Pp_GH2, Rs_N1, Rs_14, Rs_5, Pp_Cr, and *w*Ppe in terms of essential features, including the absence of CI (cytoplasmic incompatibility) genes *cifA* and *cifB*, plasmid-associated genes, and WO prophages or phage-like proteins, that are linked with *Wolbachia* phenotypes, such as CI (Beckmann and Fallon, 2013; Metcalf et al., 2014; Papafotiou et al., 2011; Siozios et al., 2013). Unlike other *Wolbachia* from the L supergroup, *w*Het had the gene *thiE* encoding thiamine phosphate synthase. Strain *w*Het harbored several genes associated with heme pathways, including *hemA, hemB, hemC, hemE, hemF, hemH, ctaA,* and *ctaB*, which were also present in *w*Ppe and *w*Tex, except for *hemH* being absent in *w*Tex. These findings suggest that *w*Het may serve as an exogenous source for heme in the host nematode as nematodes are thought to be unable to synthesize heme (Rao et al., 2005). Among several nutrients (thiamine, biotin riboflavin, and heme) that were proposed as candidate nutrients supplied by other *Wolbachia* strains as a means to maintain *Wolbachia* through a weak or facultative mutualism (Brown et al., 2016; Foster et al., 2005; Gill et al., 2014; Moriyama et al., 2015), only the heme pathway appeared to be complete in *w*Het. However, uncertainty remains about the major functions of *w*Het with respect to its host, given that our genomic analysis showed that *w*Het overall had a large number (506) of predicted proteins with unknown functions.

At the pathway level, analysis showed some differences in functional enrichment. For example, GO-figure plots, based on semantic similarity, depicted similar functional patterns of *w*Het’s biological process enrichment with nematode *Wolbachia* supergroups (C and L). However, when compared individually to each known member of the PPN supergroup from each host (*Wolbachia* strains from *P. penetrans* and *R. similis,* and *w*Tex), *w*Het showed more relatedness in gene repertoire and associated biological processes to *w*Tex, which comprises a sample collected from a nematode community about which there remains little host or symbiont biological information (Weyandt et al., 2022). Notably, the gene ontology enrichment analysis showed differences in significantly enriched GO terms in *w*Het compared to supergroups from filarial nematodes (C, D, and J) with a notable enrichment in nitrogen metabolic processes. We speculate that this enrichment could reflect a biological role for *w*Het related to its nematode lifestyle in nitrogen-limited conditions of root feeding; however, this hypothesis would require experimental investigation.

Strain *w*Het’s GC content and genome size were similar to that of other PPN *Wolbachia*, as expected. Furthermore, the plot GC content and genome size, including strain *w*Het, shows a positive correlation between GC content and genome size across *Wolbachia* strains. Although endosymbionts usually have reduced genome size and metabolic capabilities compared to their free-living relatives (Newton et al., 2016; Newton and Bordenstein, 2011), here, the reduced GC content in *w*Het and PPN-type *Wolbachia* compared to most A and B group strains may be due to a stronger pattern of vertical transmission in PPN-type *Wolbachia* leading to higher levels of bottleneck and drift with an underlying AT-mutational bias and the inability to purge mutations under strong bottleneck (Brown et al., 2015; Moran and Bennett, 2014; Moran and Plague, 2004; Renoz et al., 2022). Phylogenetic analyses thus far showing longer branches in this clade seem to support this stronger vertical transmission hypothesis.

Acknowledging that some of the PPN *Wolbachia* genome lengths included in this study may be underestimated due to varying degrees of incompleteness, our data suggest clear differences in length between the complete *w*Het genome and those of other supergroups. The reduced genome length in *w*Het and PPN-type *Wolbachia* compared to A and B group strains may be due to the gradual erosion and elimination of non-functional sequences that become redundant within the intracellular environment where the host provides many metabolites directly to the symbiont, accelerating genome size loss due to genetic drift causing fixation of irreversible deleterious mutations (Bobay and Ochman, 2017). Although the genome repertoire and GC content of *w*Het and others in this supergroup were more similar to supergroups C and D, which are obligate mutualists from filarial nematodes, compared to supergroups A and B, which are mostly CI-inducing *Wolbachia* strains, the scant data on the PPN *Wolbachia* prevalence thus far suggest some supergroup L strains are not at 100% prevalence (Wasala et al., 2019) while others may be at 100% prevalence (Haegeman et al., 2009). However, the lack of previous reports of *Wolbachia*-like endosymbionts in Heteroderidae nematodes suggests *w*Het, such as *w*Ppe, is likely not an obligate mutualist. Nevertheless, based on the trend toward shorter genome length and lower GC content similar to C and D supergroup *Wolbachia* strains, it is possible that PPN *Wolbachia* strains could be facultative mutualists.

Although we found a high-quality genome supporting the new strain *w*Het in *H. schachtii,* confirmation of the host will require additional fluorescent *in situ* hybridization (FISH) for isolates of sugarbeet nematode or alfalfa cyst nematode. Furthermore, to assess this symbiont’s distribution, a broad sampling of *H. schachtii* and *H. medicaginis*-like relatives will be important. Predicted genes with unknown functions in *w*Het, which were abundant in its accessory genome, limited the ability of GO and pathway analysis to distinguish potential functions. Future annotation of such genes may uncover important aspects of this host–symbiont relationship, but investigating the function of *w*Het in the host will require future experiments, such as symbiont-clearing assays and RNA-seq analysis to examine how specific gene pathways are altered in response to *w*Het infection.

## Data Availability

SRA data and whole genome sequencing assemblies analyzed in this study were accessed through NCBI Accessions (https://www.ncbi.nlm.nih.gov). The datasets supporting the conclusions of this article are available in the NCBI repository, BioProject PRJNA767548, BioSamples SAMN21928827, and the final assembled genome was deposited under BioProject PRJNA1148971.

## Footnotes

^1^https://github.com/BioInfoTools/BBMap/blob/master/sh/pileup.sh

^2^http://bioinformatics.psb.ugent.be/webtools/Venn/

^3^https://github.com/lyijin/topGO_pipeline/
