# Supplementary material for: Discovery of a novel Wolbachia in Heterodera expands nematode host distribution
Source: Front Microbiol. 2024 Sep 25;15:1446506. doi: 10.3389/fmicb.2024.1446506 (PMC11461310; doi:10.3389/fmicb.2024.1446506)
Supplement: Supplementary file 1 [file Data_Sheet_1.pdf]

Supplementary Material

Discovery of a novel *Wolbachia* in *Heterodera* expands nematode host distribution

Taranjot Kaur[1], Amanda M.V. Brown[1]*

*** Correspondence:** Amanda M.V. Brown: amanda.mv.brown@ttu.edu

Supplementary Figures

A **B**

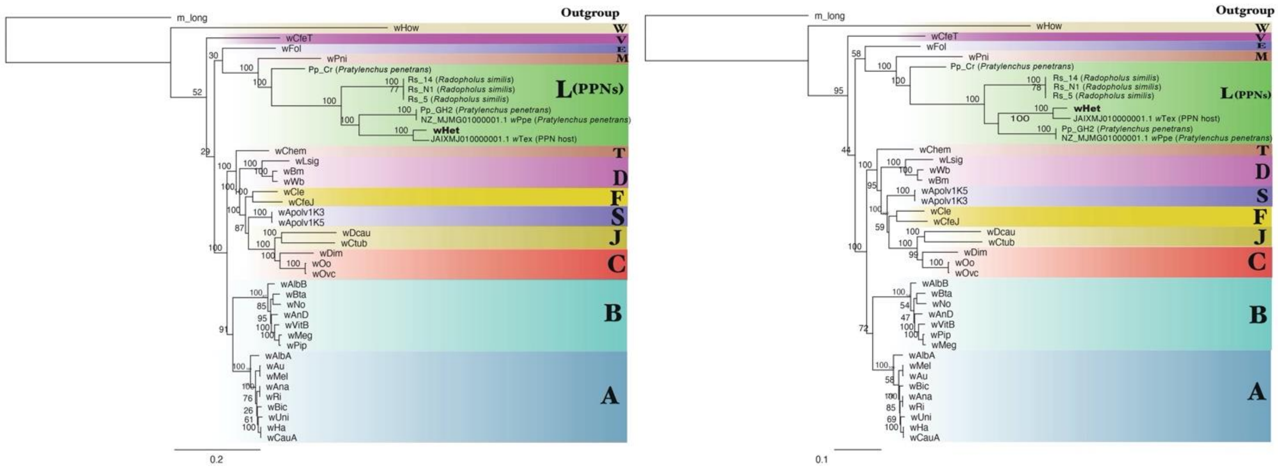

Supplementary Figure 1. Phylogenetic trees of *Wolbachia* strains and outgroup generated from an alignment of 37 core genes (32,748 nucleotide positions) with (A) No gaps removed (B) All gaps removed, performed in RAxML with the GTR+Gamma model with support from 100 bootstrap replicates shown on branches. The clade including *w*Het is shown in bold. Letters represent supergroups A (insects), B (insects), C (filarial nematodes), D (filarial nematodes), E, F, J, M, S, T, V, W, and supergroup L (plant-parasitic nematodes).

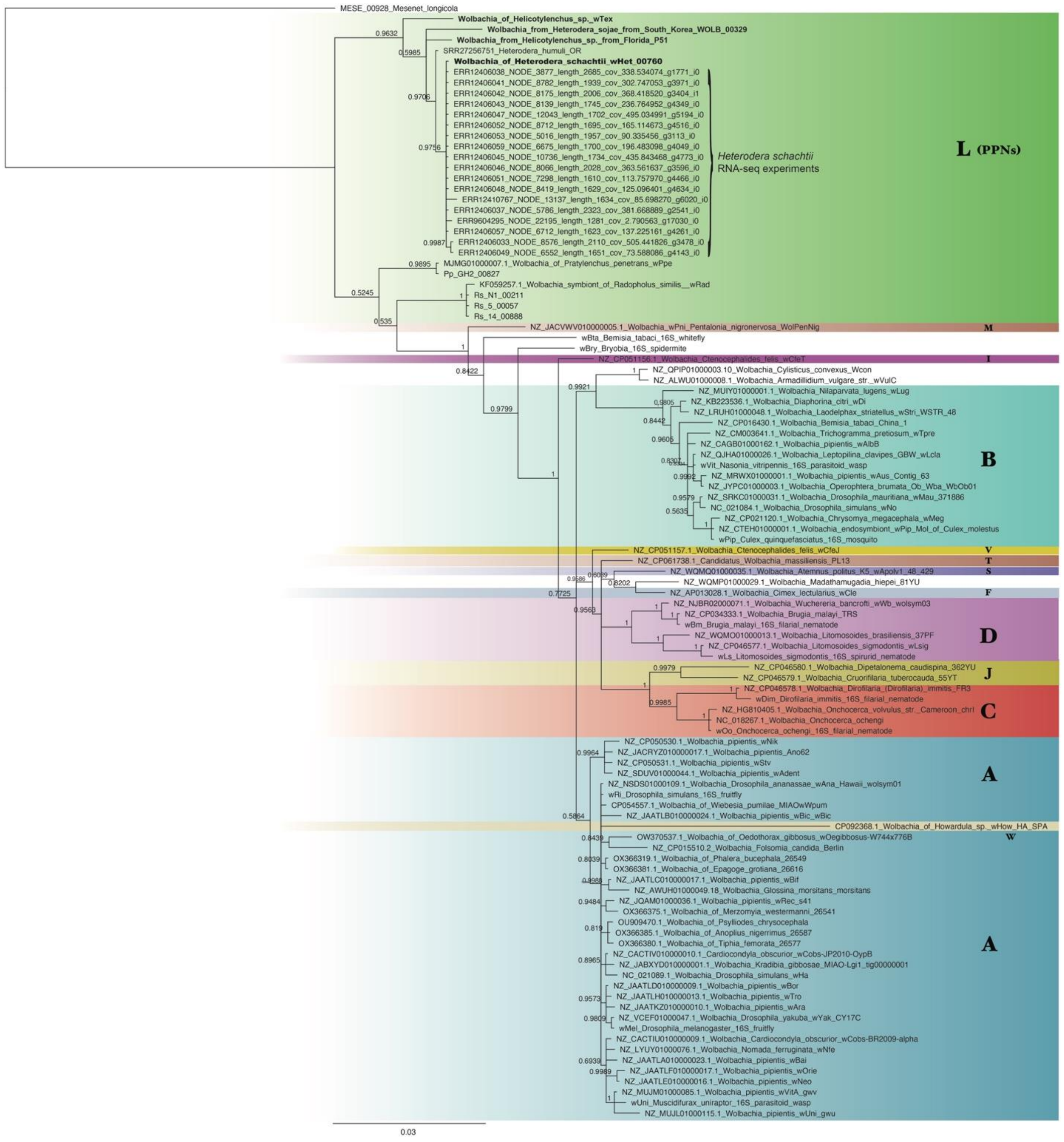

Supplementary Figure 2. Phylogenetic tree of *Wolbachia* strains and *Candidatus* Mesenet longicola outgroup generated by MrBayes from a 1530 bp alignment of the 16S rRNA gene, with posterior probabilities shown on branches. The clade including *w*Het is shown in bold. Letters represent supergroups A (insects), B (insects), C (filarial nematodes), D (filarial nematodes), F, M, S, T, and supergroup L (plant-parasitic nematodes).

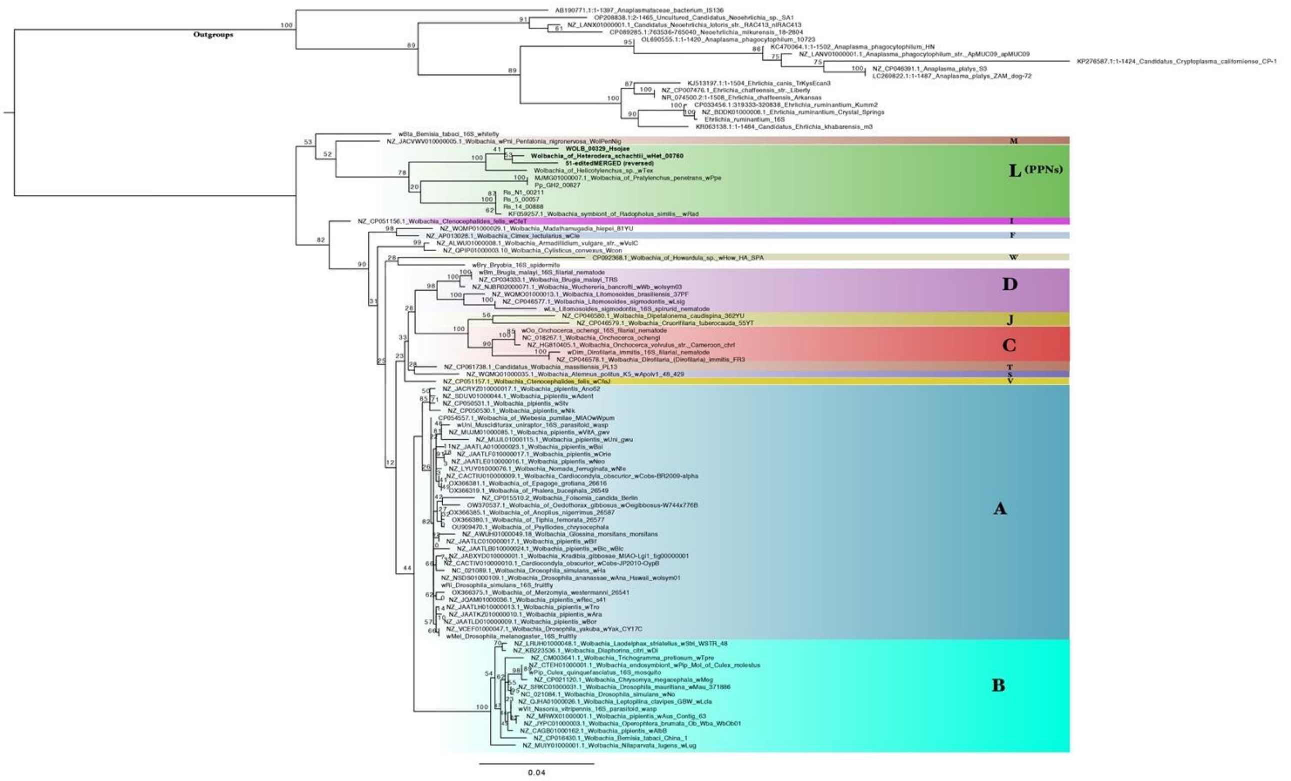

Supplementary Figure 3. Phylogenetic tree of *Wolbachia* strains and Anaplasmataceae outgroups generated by RAxML with the GTR+Gamma model from a 1548 bp alignment of the 16S rRNA gene, with support from 100 bootstrap replicates shown on branches. The clade including *w*Het is shown in bold. Letters represent supergroups A (insects), B (insects), C (filarial nematodes), D (filarial nematodes), F, M, S, T, and supergroup L (plant-parasitic nematodes).

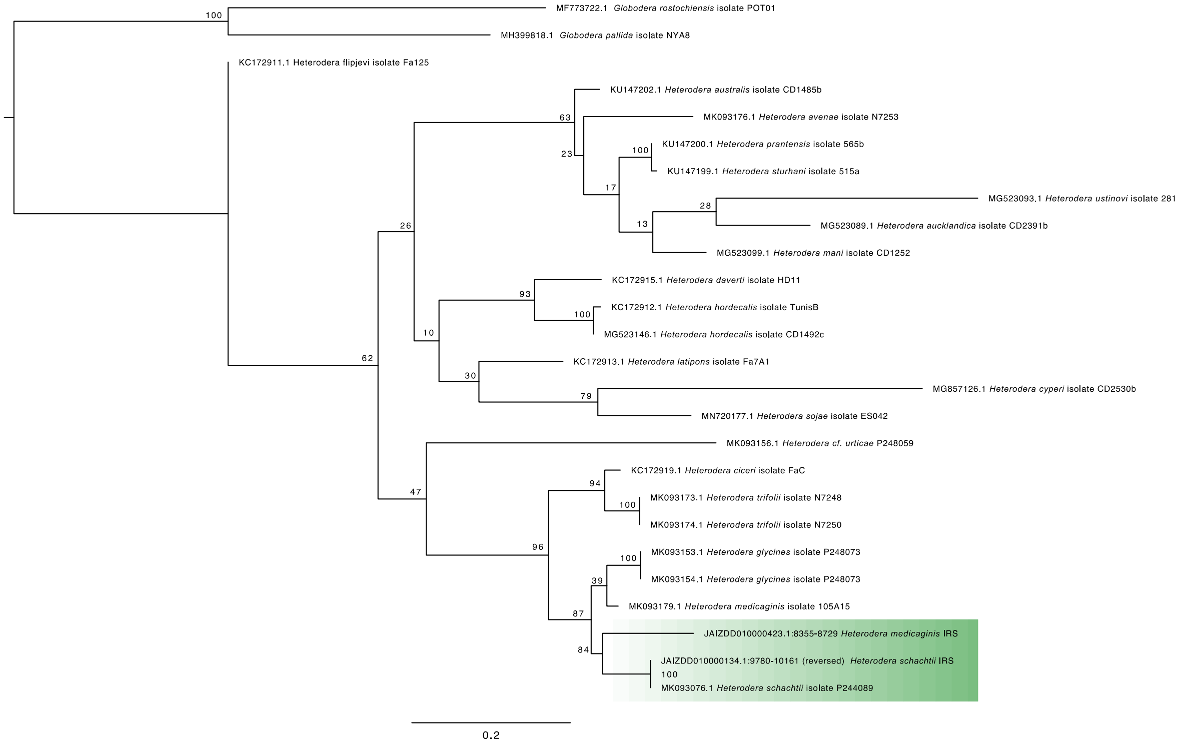

Supplementary Figure 4. Phylogenetic tree for the COI gene alignment from outgroups and *Heterodera* spp. including COI genes recovered from the *H. schachtii* IRS assembly performed in RAxML with the GTR+Gamma model, with support from 100 bootstrap replicates shown on branches.

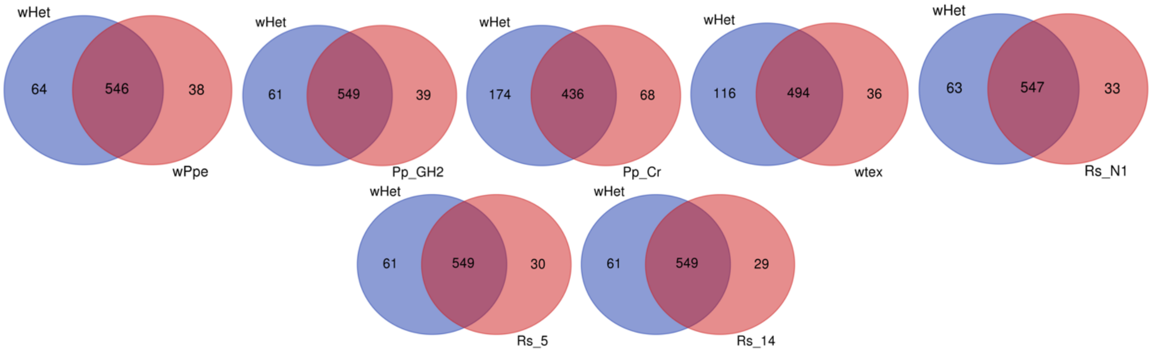

Supplementary Figure 5. Venn diagrams showing number of shared and unshared genes among supergroup L members, and *w*Het.

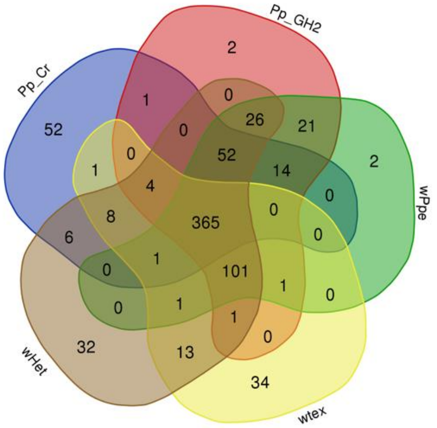

Supergroup L (*Pratylenchus* spp.)

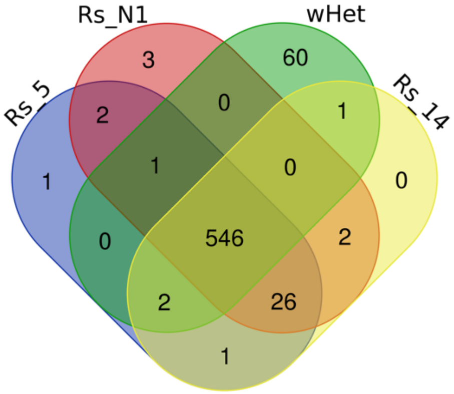

Supergroup L (*Radophulus* spp.)

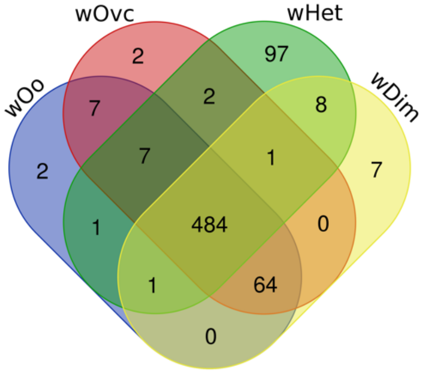

Supergroup C

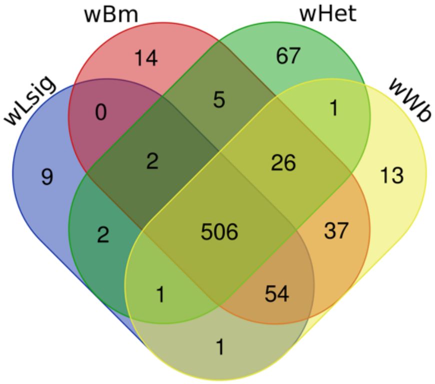

Supergroup D

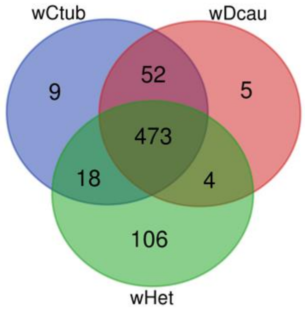

Supergroup J

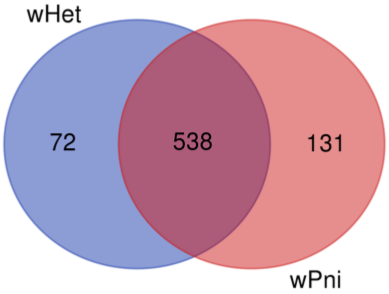

Supergroup M

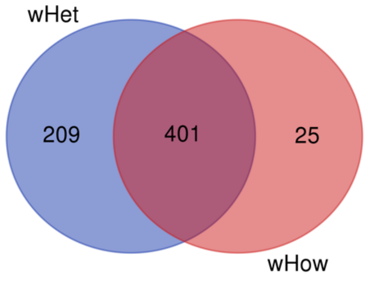

Supergroup W

Supplementary Figure 6. Venn diagrams showing number of shared and unshared genes among different *Wolbachia* from C, D, J, M, L, W supergroups (nematodes).

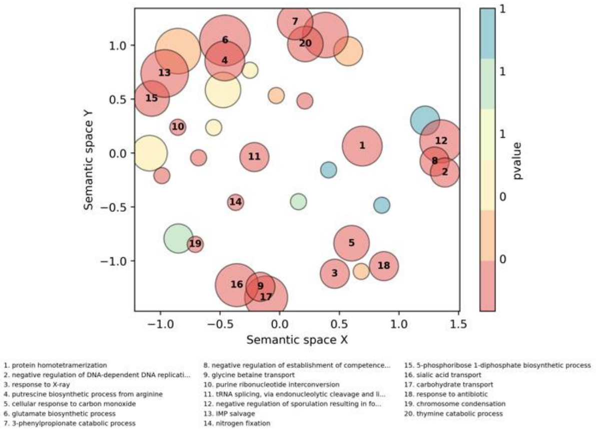

(A) supergroup A

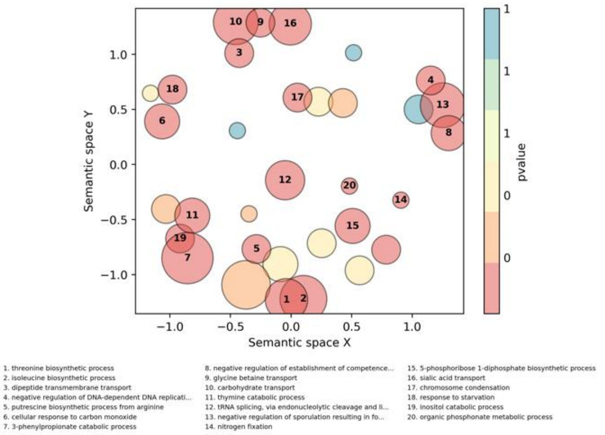

(B) supergroup B

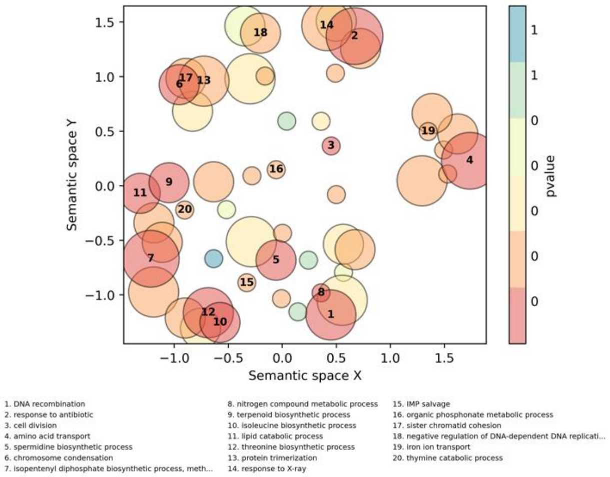

(C) supergroup C

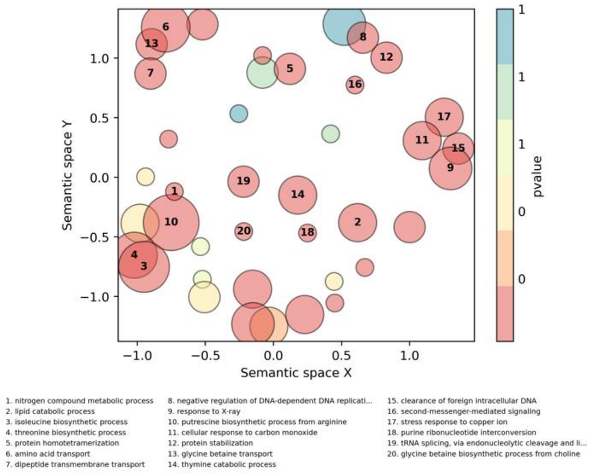

(D) supergroup D

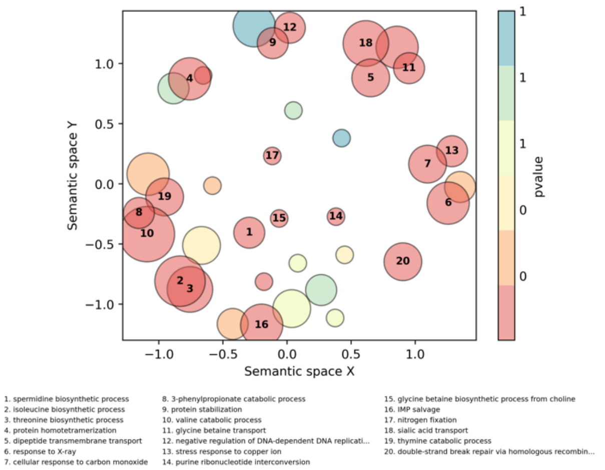

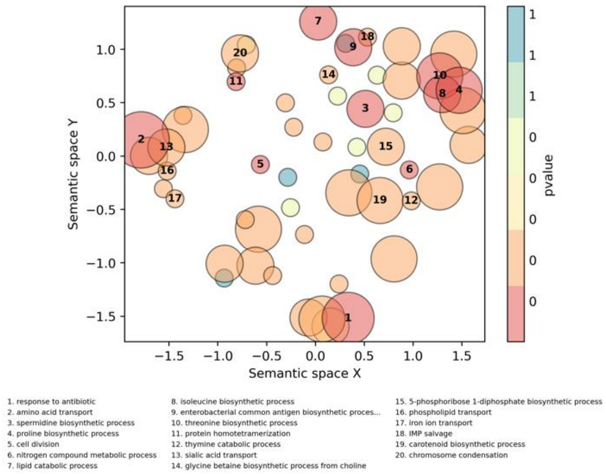

(E) supergroup F

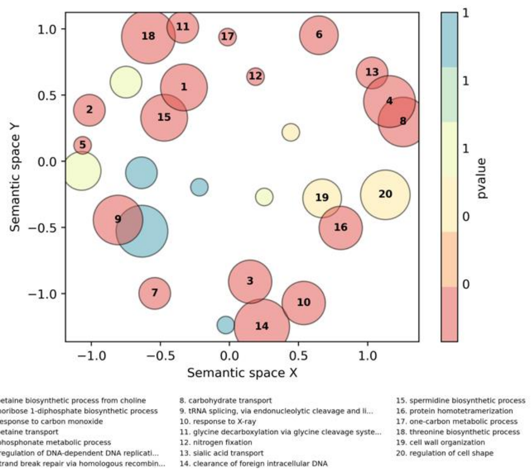

(F) supergroup J

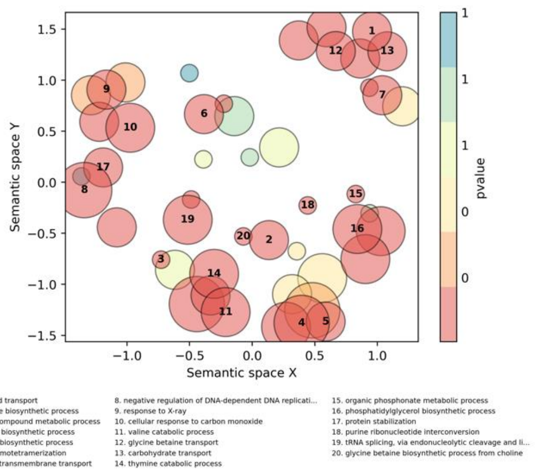

(G) Supergroup L

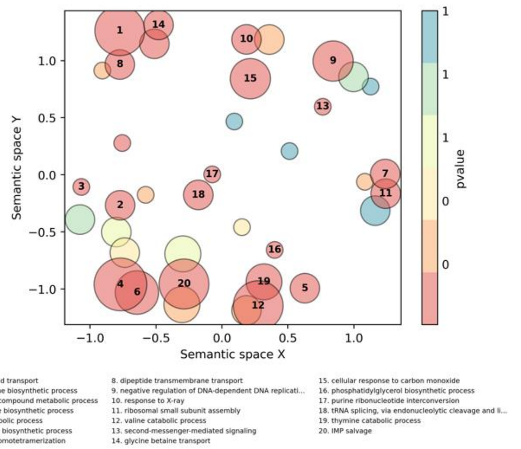

(H) Supergroup S

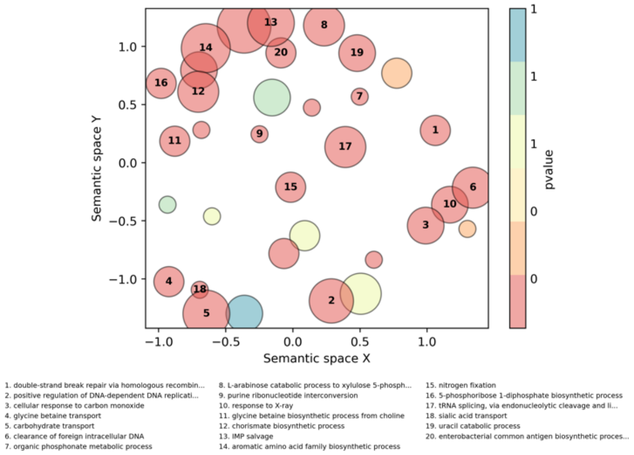

(I) supergroup T

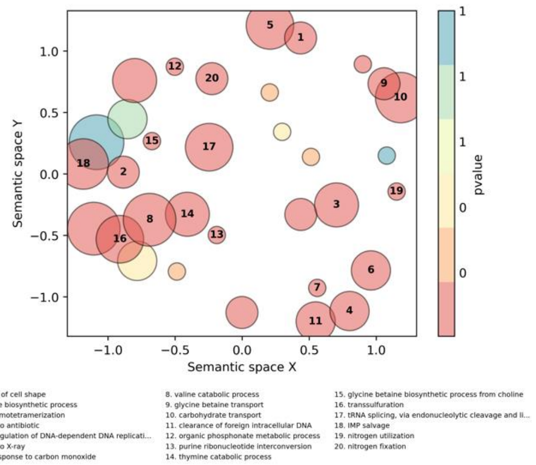

(J) *P. penetrans Wolbachia* strains

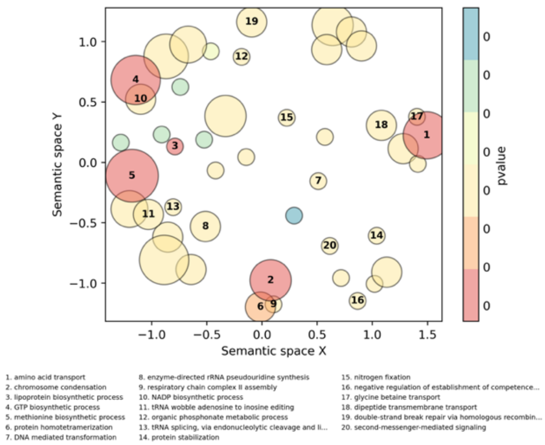

(K) ***R. similis Wolbachia*** **strains** **(L)** ***w*****Tex**

Supplementary Figure 7. Patterns of significantly enriched Gene Ontology (GO) categories of *w*Het genes compared to **(A)** supergroup A, **(B)** supergroup B, **(C)** supergroup C, **(D)** supergroup D, **(E)** supergroup F, **(F)** supergroup J, **(G)** supergroup L, **(H)** supergroup S, **(I)** Supergroup T (**J)** *P. penetrans Wolbachia* strains (supergroup L), **(K)** *R. similis Wolbachia* strains (supergroup L), **(L)** *w*Tex (supergroup L). The color of the node represents the p-value for the term. Numbers 1-20 indicate the top 20 most significant (lowest p-values) for GO terms based on topGO outputs.

Supplementary Tables

Supplementary Table 1: Presence/absence of *Wolbachia* in whole genome sequencing (WGS) assemblies and SRA reads available for *Meloidogyne* spp. in NCBI.

Organism Name	Genbank acession	Submitter	WGS project	Sequencing platform	Sequence Length	Number of scaffolds	Presence of *Wolbachia*
Meloidogyne incognita	GCA_036172945.1	Huazhong Agricultural University, China	JARQAC01	Oxford Nanopore PromethION	225	77	No
Meloidogyne graminicola	GCA_036172905.1	Huazhong Agricultural University, China	JARPZY01	PacBio Sequel	39.18	18	No
Meloidogyne javanica	GCA_036172935.1	Huazhong Agricultural University, China	JARQAA01	PacBio Sequel	301.18	97	No
Meloidogyne chitwoodi	GCA_015183035.1	Oregon State University, USA	JACZZP01	PacBio Sequel RSII	47.48	30	No
Meloidogyne arenaria	GCA_036172955.1	Huazhong Agricultural University, China	JARQAB01	PacBio Sequel	315	475	No
Meloidogyne hapla	GCA_000172435.1	North Carolina State University, USA	ABLG01		53.01	3450	No
Meloidogyne exigua isolate Mex1	GCA_018905775.1	Institut de Recherche Pour le Developpement, France	JAGUQR01	Oxford Nanopore Technology; Illumina	42.1	206	No
Meloidogyne enterolobii	GCA_903994135.1	INRAE	CAJEWN01	PacBio RS	240.1	4437	No
Meloidogyne floridensis isolate SJF1	GCA_003693605.1	University of Hull, England	RCFN01	Illumina HiSeq	74.85	8887	No
Meloidogyne luci strain SI-Smartno	GCA_902706615.1	Wellcome Sanger Institute, UK	CACSLI01		209.2	327	No
Meloidogyne incognita isolate WHF4-1	GCA_036172895.1	Huazhong Agricultural University, China	JARPZX01	PacBio Sequel	213.3	129	No
Meloidogyne incognita strain Kmmt_Gs004	GCA_014132215.1	Kazusa DNA Research Institute, Japan	BLLR01	PacBio Sequel	193.2	374	No
Meloidogyne incognita	GCA_900182535.1	Genoscope CEA, France	FXSY01		183.5	12,091	No
Meloidogyne incognita isolate W1	GCA_003693645.1	University of Hull, England	RCFL01	Illumina HiSeq	122	333,351	No
Meloidogyne graminicola isolate MgF1-1	GCA_036172845.1	Huazhong Agricultural University, China	JARPZT01	PacBio Sequel	45.8	28	No

Meloidogyne graminicola isolate VN-18	GCA_014773135.1	Consultative Group for International Agricultural Research Program on rice-agrifood systems	JABEBT01	Illumina HiSeq; Oxford Nanopore Technology	41.55	283	No
Meloidogyne graminicola isolate IARI	GCA_002778205.2	ICAR-IARI, New Delhi, INDIA	NXFT02	PacBio Sequel	36.83	513	No
Meloidogyne javanica isolate VW4	GCA_034785575.1	University of Hull, England	JARDRP01	PacBio Sequel; Oxford Nanopore PromethION; Illumina HiSeq	150.5	61	No
Meloidogyne javanica isolate LongYF2-1	GCA_036172855.1	Huazhong Agricultural University, China	JARPZV01	PacBio Sequel	288.4	71	No
Meloidogyne javanica isolate VW4	GCA_003693625.1	University of Hull, England	RCFK01	Illumina HiSeq	150.3	34,316	No
Meloidogyne javanica	GCA_900003945.1		CEWN01		235.8	31,341	No
Meloidogyne chitwoodi isolate Roza	GCA_015183025.1	Northwest Potato Research Consortium	JACZZN01	PacBio Sequel RSII	47.73	38	No
Meloidogyne chitwoodi isolate Race2	GCA_015183065.1	Northwest Potato Research Consortium	JACZZO01	PacBio Sequel RSII	46.92	39	No
Meloidogyne arenaria isolate SYMaF1-2	GCA_036172835.1	Huazhong Agricultural University, China	JARPZW01	PacBio Sequel	311.8	548	No
Meloidogyne arenaria isolate A2-J Honshu_01	GCA_017562155.1	RIKEN	JAEEAS01	PacBio RSII	281.7	1430	No
Meloidogyne arenaria isolate A2-O Okinawa_01	GCA_003133805.1	RIKEN	QEUI01	PacBio	284	2,223	No
Meloidogyne arenaria	GCA_900003985.1	Genoscope CEA, France	CEWM01	Illumina HiSeq	258.1	26,196	No
Meloidogyne arenaria isolate HarA	GCA_003693565.1	University of Hull, England	RCFJ01		163.7	46,436	No
Meloidogyne enterolobii	GCA_903797545.1	INRAE	CAIGJL01	PacBio	240.1	4,437	No
Meloidogyne enterolobii isolate L30	GCA_003693675.1	University of Hull, England	RCFM01	Illumina HiSeq	163	42,008	No
Meloidogyne floridensis	GCA_000751915.1	BANG	CCDZ01		96.67	58,696	No
Meloidogyne incognita strain Morelos	GCA_000180415.1	Genoscope CEA, France	CABB01		82.1	9,538	No
Meloidogyne incognita isolate Gr-Nem-00857	GCA_034704635.1	LOEWE Centre for Translational Biodiversity Genomics	JAQIYG01	Illumina NovaSeq	89.13	40,268	No

Supplementary Table 2: Presence/absence of *Wolbachia* in whole genome sequencing (WGS) assemblies and SRA reads available for *Globodera* spp. in NCBI.

Organism Name	Genbank acession	Submitter	WGS project	Sequencing platform	Sequence Length	Number of scaffolds	Presence of *Wolbachia*
Globodera pallida isolate D383	GCA_020449905.1	Wageningen University and Research, Netherlands	JAIXHY01	PacBio; Illumina	113.2	163	No
Globodera rostochiensis isolate L19	GCA_018350325.1	Wageningen University and Research, Netherlands	JAEVLN01	PacBio RS; Illumina NovaSeq	92.7	88	No
Globodera ellingtonae isolate Ge_1	GCA_001723225.1	Oregon State University, USA	MEIZ01	Illumina MiSeq; Illumina HiSeq; PacBio	106	2,246	No
Globodera pallida isolate Newton	GCA_023343765.1	University of St Andrews, Scotland	JAGDFN01	PacBio Sequel; Illumina HiSeq	119.6	173	No
Globodera pallida isolate Lindley	GCA_000724045.1	WTSI	CBXT01		123.6	6,873	No
Globodera rostochiensis isolate L22	GCA_018350315.1	Wageningen University and Research, Netherlands	JAEVLO01	PacBio RS; Illumina NovaSeq	101.6	135	No
Globodera rostochiensis isolate Ro1	GCA_900079975.1	BANG	FKKZ01		95.9	4,281	No

Supplementary Table 3: Quast assembly statistics for the *w*Het genome.

Assembly	*w*Het
# contigs (>= 0 bp)	1
# contigs (>= 1000 bp)	1
# contigs (>= 5000 bp)	1
# contigs (>= 10000 bp)	1
# contigs (>= 25000 bp)	1
# contigs (>= 50000 bp)	1
Total length (>= 0 bp)	1079546
Total length (>= 0 bp)	1079546
Total length (>= 5000 bp)	1079546
Total length (>= 10000 bp)	1079546
Total length (>= 25000 bp)	1079546
Total length (>= 50000 bp)	1079546
# contigs	1
Largest contig	1079546
Total length	1079546
GC (%)	32.59
N50	1079546
N75	1079546
L50	1
L75	1
# N's per 100 kbp	0.56
